# Comparing Alternative Single-Step GBLUP Approaches and Training Population Designs for Genomic Evaluation of Crossbred Animals

**DOI:** 10.3389/fgene.2020.00263

**Published:** 2020-04-09

**Authors:** Amanda B. Alvarenga, Renata Veroneze, Hinayah R. Oliveira, Daniele B. D. Marques, Paulo S. Lopes, Fabyano F. Silva, Luiz F. Brito

**Affiliations:** ^1^Department of Animal Sciences, Purdue University, West Lafayette, IN, United States; ^2^Department of Animal Science, Federal University of Viçosa, Viçosa, Brazil; ^3^Department of Animal Biosciences, Centre for Genetic Improvement of Livestock, University of Guelph, Guelph, ON, Canada

**Keywords:** crossbred performance, ssGBLUP, simulated dataset, training population design, WssGBLUP

## Abstract

As crossbreeding is extensively used in some livestock species, we aimed to evaluate the performance of single-step GBLUP (ssGBLUP) and weighted ssGBLUP (WssGBLUP) methods to predict Genomic Estimated Breeding Values (GEBVs) of crossbred animals. Different training population scenarios were evaluated: (SC1) ssGBLUP based on a single-trait model considering purebred and crossbred animals in a joint training population; (SC2) ssGBLUP based on a multiple-trait model to enable considering phenotypes recorded in purebred and crossbred training animals as different traits; (SC3) WssGBLUP based on a single-trait model considering purebred and crossbred animals jointly in the training population (both populations were used for SNP weights' estimation); (SC4) WssGBLUP based on a single-trait model considering only purebred animals in the training population (crossbred population only used for SNP weights' estimation); (SC5) WssGBLUP based on a single-trait model and the training population characterized by purebred animals (purebred population used for SNP weights' estimation). A complex trait was simulated assuming alternative genetic architectures. Different scaling factors to blend the inverse of the genomic (**G**^−1^) and pedigree (${\text{A}}_{22}^{-1}$) relationship matrices were also tested. The predictive performance of each scenario was evaluated based on the validation accuracy and regression coefficient. The genetic correlations across simulated populations in the different scenarios ranged from moderate to high (0.71–0.99). The scenario mimicking a completely polygenic trait ($h_{QTL}^{2}=$ 0) yielded the lowest validation accuracy (0.12; for SC3 and SC4). The simulated scenarios assuming 4,500 QTLs affecting the trait and $h_{QTL}^{2}=h^{2}$ resulted in the greatest GEBV accuracies (0.47; for SC1 and SC2). The regression coefficients ranged from 0.28 (for SC3 assuming polygenic effect) to 1.27 (for SC2 considering 4,500 QTLs). In general, SC3 and SC5 resulted in inflated GEBVs, whereas other scenarios yielded deflated GEBVs. The scaling factors used to combine **G**^−1^ and ${\text{A}}_{22}^{-1}$ had a small influence on the validation accuracies, but a greater effect on the regression coefficients. Due to the complexity of multiple-trait models and WssGBLUP analyses, and a similar predictive performance across the methods evaluated, SC1 is recommended for genomic evaluation in crossbred populations with similar genetic structures [moderate-to-high (0.71–0.99) genetic correlations between purebred and crossbred populations].

## Introduction

Crossbreeding schemes are paramount for some livestock production systems in enabling the exploitation of complementarity among genetically-divergent breeds and heterosis effects (Wei and van der Werf, 1994). In tropical countries, crosses between two cattle sub-species are widely used to combine climatic adaptability (e.g., from *Bos taurus indicus*; Zebu breeds) and productive performance (e.g., from *Bos taurus taurus*; Taurine breeds) traits (Gregory and Cundiff, 1980; Mendonça et al., 2019). Genetic selection is performed on purebred animals in these production systems, aiming to optimize the performance of crossbred progeny. However, this poses various challenges to the breeding programs. For instance, there are large differences in additive and non-additive genetic parameters in traits measured in purebred or crossbred animals (Bijma and van Arendonk, 1998), which might constrain the pooling of all animals into a single training population for genomic analysis (Ribeiro et al., 2019). However, the large majority of livestock breeding programs do not account for non-additive genetic effects when estimating breeding values, and most economically important traits in livestock are not largely influenced by non-additive genetic effects (Varona et al., 2018).

Recording large-scale phenotypes on crossbred animals raised in commercial herds is usually a challenge, especially for hard- or expensive-to-measure traits, such as individual feed intake (Ibánêz-Escriche et al., 2009). Over time, several methods to perform genetic evaluations accounting for purebred and crossbred information have been proposed (Bijma and van Arendonk, 1998; Nayee et al., 2016; Junqueira et al., 2017). For instance, Wei and van der Werf (1994) proposed a model of breeding value prediction for both purebred and crossbred animals that maximizes the genetic response in crossbred animals, even for unknown, or inappropriate values of correlations of purebred and crossbred performances, and crossbreeding heritability. However, in the genomic era, Ibánêz-Escriche et al. (2009) have suggested that genomic information can increase the response to selection for crossbred performance even when selecting only purebred animals.

Genomic selection (Meuwissen et al., 2001) has been proven to be a useful tool to increase genetic gain, especially for difficult or expensive-to-measure and/or low-heritability traits. In this context, several methods have been proposed to calculate Genomic Estimated Breeding Values (GEBV) for livestock, such as the single-step Genomic Best Linear Unbiased Prediction (ssGBLUP; Misztal et al., 2009; Aguilar et al., 2010; Christensen and Lund, 2010). The ssGBLUP enables combining the pedigree-based relationship matrix (**A**) with the genomic relationship matrix (**G**) into a hybrid matrix (**H**). This increases the accuracy and reduces the prediction bias of GEBVs when compared to those yielded from multi-step genomic predictions (Aguilar et al., 2010; Lourenco et al., 2015; Guarini et al., 2018). Recent studies have evaluated the use of purebred information to predict crossbred performance using the ssGBLUP method (Lourenco et al., 2016; Tusell et al., 2016; Pocrnic et al., 2019). In this context, Lourenco et al. (2016), using simulated crossbred pig datasets, concluded that the highest GEBV accuracies were attained when using a training population combining both purebred and crossbred animals' datasets. However, the ssGBLUP assumes equal variances for all Single Nucleotide Polymorphisms (SNPs), which may not be the most appropriate assumption from a biological point of view (Meuwissen et al., 2001; VanRaden, 2008; Goddard and Hayes, 2009). In a recent study, Porto-Neto et al. (2014) reported that nine out of ten traits evaluated were influenced by major genes. Consequently, methods that account for locus-specific variance (e.g., weighted ssGBLUP, WssGBLUP; Zhang et al., 2016) have been proposed. The main aim of these methods is to increase the predictive performance of GEBVs using computationally efficient tools that can be easily implemented in commercial breeding programs. In the WssGBLUP method, different SNP weights are used when calculating the **G** matrix.

The WssGBLUP has been successfully applied to several genomic prediction studies (Zhang et al., 2016; Lourenco et al., 2017; Guarini et al., 2019). However, to our best knowledge, there are no reports evaluating the prediction ability of WssGBLUP in crossbred animals, especially in F1 populations. Therefore, we aimed to compare the predictive performance of ssGBLUP and WssGBLUP using different training populations (based on purebred and/or crossbred animals) and alternative statistical models (single- or multiple-trait). One alternative for evaluating the predictive performance of genomic models is comparing GEBVs and True Breeding Values (TBVs). However, in practice, the TBVs are usually unknown and therefore simulated datasets can be advantageous when comparing models and genomic prediction approaches. In this context, we evaluated five simulated scenarios mimicking beef cattle populations (two purebred lines and four F1 populations), in which the trait under evaluation differed in terms of the number of Quantitative Trait Loci (QTLs) and the trait heritability (*h*^2^) explained by them ($h_{QTL}^{2}$). Furthermore, the impact of the genetic distance between training and validation populations used in the crossbreeding scheme was also investigated.

## Materials and Methods

Only (computationally) simulated datasets were used in this study. Therefore, the approval of an Institutional Animal Care and Use Committee was not required.

### Simulated Population

Datasets of purebred and crossbred animals were simulated based on a beef cattle production system. The purebred populations were simulated to mimic *Bos taurus indicus* (Line1; Zebu cattle) or *Bos taurus taurus* (Line2; Taurine cattle) animals. Crossbred animals (F1) were originated from the crossing between females from Line1 and males from Line2. Phenotypes and TBVs were simulated for a trait with a *h*^2^ equal to 0.33 and phenotypic variance equal to 0.13. This was done to mimic the trait residual feed intake (RFI; an indicator of feed efficiency), which is a very important trait in beef cattle breeding programs (Branco et al., 2014) and has a similar genetic architecture compared to many other economically important (quantitative) traits in livestock.

**Figure 1:**
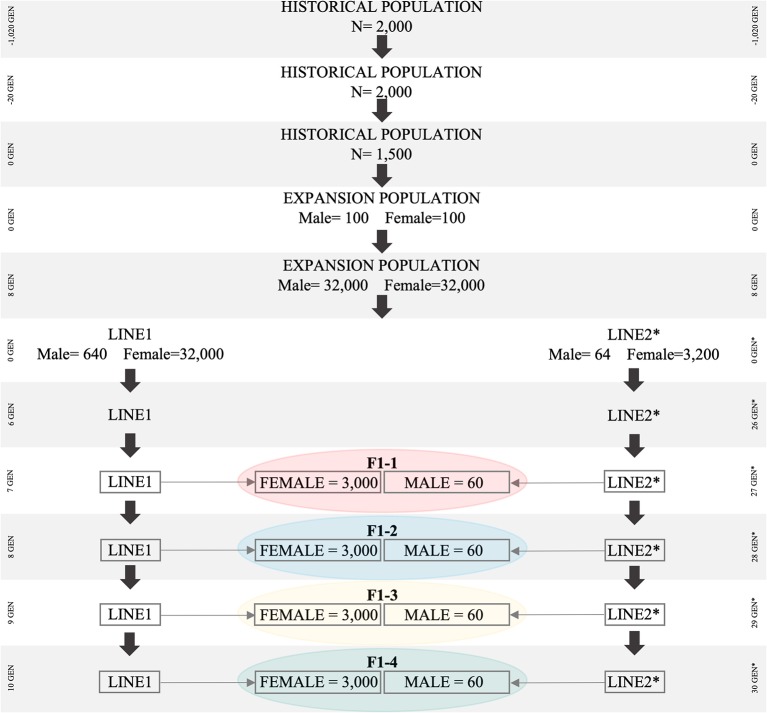
Simulated population scheme representing bottleneck in historical population, breed differentiation, and origin of F1 for all simulated scenarios. The *Bos taurus indicus* population is represented by Line1, *Bos taurus taurus* is represented by Line2.

The historical population consisted of 1,020 generations (Figure 1). During the first 1,000 generations (i.e., from generation −1,020 to generation −20), 2,000 individuals (1,000 males and 1,000 females) were randomly mated (Brito et al., 2011; Lourenco et al., 2016). From generation −19 to generation zero, a first “*bottleneck”* (i.e., population reduction) was created by reducing the total number of individuals from 2,000 to 1,500 (750 males and 750 females), which were also randomly mated. Thereafter, a second “*bottleneck”* was created by randomly sampling 100 males and 100 females from generation zero (1,500 individuals) of the historical population. These 200 individuals were used to create the expansion population (POP) containing 64,000 individuals. The population reductions (“*bottlenecks”*) were simulated to create an initial level of linkage disequilibrium (LD), which will be further explained.

Animals in POP were subjected to random selection, mating, and culling for eight generations. To increase the number of animals in POP, we assumed that each female had five offspring, with the same proportion of males and females. At the end of the eighth generation, 64,000 animals were available in POP, which was then used to create Line1 and Line2. Line1 was developed based on 32,000 females and 640 males, and Line2 was developed based on 3,200 females and 64 males; all of them were randomly selected from the eighth generation of POP. In subsequent generations of Line1 and Line2, each female had one offspring (with the same probability of being male or female), and the replacement ratio for sires and dams was 0.60 and 0.20, respectively. Selection and culling in both Line1 and Line2 were performed based on the lowest and highest Estimated Breeding Values (EBVs), respectively. EBVs were estimated based on the Best Linear Unbiased Prediction method (Henderson, 1975), through an Animal Model and considering the True Additive Genetic Variance. After 10 generations in Line1 (*Bos taurus indicus*), and 30 in Line2 (*Bos taurus taurus*), the average LD values (between adjacent SNPs) were similar to those reported for *Bos taurus indicus* (*r*^2^ = 0.20) and *Bos taurus taurus* (*r*^2^ = 0.33) (Villa-Angulo et al., 2009). Both LD values were assessed in the last generation using the distance between SNPs up to 0.05 cM.

The F1 population originated from the random mating of 3,000 females from Line1 with 60 males from Line2. A total of four F1 populations were created and they differed with regards to the parental generation used in the crossbreeding scheme. Parental animals of the F1 populations were from: (i) F1-1: generations seven and 27; (ii) F1-2: eight and 28; (iii) F1-3: nine, and 29; (iv) F1-4: ten and 30; in Line1 and Line2, respectively. The differences in the generation of Line1 and Line2 (e.g., seven for Line1 and 27 for Line2) are due to the simulation scheme designed to mimic the current pattern of LD and genetic distance between Nellore and Angus, represented by Line1 and Line2, respectively.

### Simulated Genotypes

The genomic prediction was performed using simulated genotypes for animals from generations six to eight (for Line1), generations 26 to 28 (for Line2), and all F1 individuals. Animals from the last two generations of the purebred lines (i.e., generations nine and ten for Line1, and 29 and 30 for Line2) were not included in the analyses in order to maintain a genetic distance between training and validation populations (described below). The simulated genotypes consisted of 52,886 bi-allelic SNPs distributed across 29 chromosomes (autosomes), mimicking the bovine genome. The size of the whole genome was ~2,696.54 cM. The number of SNPs and the size of each chromosome was defined based on information retrieved from the Illumina Bovine 50 K Beadchip (https://support.illumina.com/downloads/bovinesnp50v2.html), as suggested by Matukumalli et al. (2009). The SNPs were evenly spaced within each chromosome and the initial allele frequency for SNPs and QTLs were equal to 0.50 in the first generation of the historical population.

Different $h_{QTL}^{2}$ and numbers of QTLs were used in this study: (i) $h_{QTL}^{2}\;$equal to zero, to represent a completely polygenic trait (SIM1); (ii) $h_{QTL}^{2}$ equal to 1/3 of the trait *h*^2^ (i.e., $h_{QTL}^{2}\;$equal to 0.11), and 198 QTLs (SIM2); (iii) $h_{QTL}^{2}$ equal to 1/3 of the trait *h*^2^ and 4,500 QTLs (SIM3); (iv) $h_{QTL}^{2}$ equal to the trait *h*^2^ (i.e., 0.33), and 198 QTLs (SIM4); (v) $h_{QTL}^{2}$ equal to the trait *h*^2^ and 4,500 QTLs (SIM5). The heritability only due to the QTL effects, $h_{QTL}^{2}$, represents the proportion of the total genetic variation of a trait that is due to a limited number of QTLs (i.e., 198 or 4,500) out of all the markers simulated. In other words, it does not indicate the complete inheritance mode of the trait, but the proportion of the total genetic variance explained by the simulated QTLs. The number of QTLs (198) was defined based on a systematic review performed for RFI in beef cattle (Duarte et al., 2019). In addition, simulations considering 4,500 QTLs were also performed, assuming that not all QTLs for RFI are currently known.

The effect of each QTL was sampled from a Gamma distribution with a shape parameter of 0.40. The mutation rate for both SNPs and QTLs was considered as 10^−5^ per generation and locus. The QTL effect captured by the SNP marker can potentially change across populations and generations due to the population-specific allele frequency and LD levels between SNP markers and QTLs. In order to minimize the effects of the simulation (starting values) in the results, ten independent replicates were carried out for each scenario. Simulations were performed using the QMSim software (Sargolzaei and Schenkel, 2009).

### Genotypic Quality Control

Genotypic quality control was performed independently for each population (Line1, Line2, and F1 populations) and replicated. The genotype quality control kept SNPs with minor allele frequency (MAF) higher 0.05, and departure from the Hardy–Weinberg Equilibrium (estimated as the difference between expected and observed frequency of heterozygous) lower than 0.15. Only common SNPs across populations were kept for further analyses. A summary of the descriptive statistics for Line1, Line2, and F1 in each scenario is shown in Table 1. Detailed descriptive statistics for each replicate are shown in the Supplementary Material (Tables S1A–S1E). The PREGSF90 software (Aguilar et al., 2014) was used to perform the genotypic quality control.

**Table 1:** Mean and standard deviation (inside parentheses) of phenotypes ($\bar{X}$), inbreeding coefficients (F), average allele A frequency (ρ_*A*_), average linkage disequilibrium (LD), and number of markers before (SNP_beforeQC_), and after (SNP_afterQC_) genotypic quality control for Line1, Line2, and F1 populations, in the different scenarios (SIM).

**SIM**	**Pop**.	**X**	**F**	**ρ_A_**	**^a^LD**	**SNP_**beforeQC**_**	**^b^SNP_**afterQC**_**
SIM1	Line1	−0.75 (0.136)	0.02 (0.009)	0.33 (0.107)	0.20 (0.005)	48,261	44,834
	Line2	−2.83 (0.131)	0.12 (0.026)	0.30 (0.125)	0.30 (0.010)		
	F1	−1.84 (0.130)	0.02 (0.013)	0.33 (0.110)	0.21 (0.007)		
SIM2	Line1	−0.72 (0.135)	0.02 (0.009)	0.34 (0.107)	0.19 (0.006)	48,241	43,995
	Line2	−2.69 (0.125)	0.13 (0.026)	0.29 (0.126)	0.31 (0.009)		
	F1	−1.76 (0.127)	0.02 (0.014)	0.33 (0.109)	0.21 (0.005)		
SIM3	Line1	−0.73 (0.136)	0.02 (0.009)	0.34 (0.107)	0.19 (0.006)	48,261	44,097
	Line2	−2.80 (0.130)	0.13 (0.026)	0.29 (0.126)	0.31 (0.017)		
	F1	−1.82 (0.130)	0.02 (0.013)	0.33 (0.110)	0.21 (0.008)		
SIM4	Line1	−0.69 (0.129)	0.02 (0.010)	0.34 (0.106)	0.19 (0.005)	48,250	42,142
	Line2	−1.98 (0.099)	0.15 (0.030)	0.28 (0.129)	0.36 (0.034)		
	F1	−1.37 (0.111)	0.02 (0.016)	0.33 (0.109)	0.22 (0.007)		
SIM5	Line1	−0.74 (0.136)	0.02 (0.009)	0.34 (0.106)	0.19 (0.006)	48,229	42,410
	Line2	−2.70 (0.120)	0.14 (0.027)	0.28 (0.129)	0.36 (0.025)		
	F1	−1.76 (0.126)	0.02 (0.015)	0.32 (0.109)	0.22 (0.007)		

*SIM, Simulated dataset; Pop., Population; SIM1, simulated dataset with heritability explained by the quantitative trait loci (h^2^_QTL_) = 0; SIM2, simulated dataset with h^2^_QTL_ = 0.11 and 198 QTLs; SIM3, simulated dataset with h^2^_QTL_ = 0.11 and 4,500 QTLs; SIM4, simulated dataset with h^2^_QTL_ = 0.33 and 198 QTLs; SIM5, simulated dataset with h^2^_QTL_ = 0.33 and 4,500 QTLs; Line1, Line1 population at six, seven and eight generations; Line2, Line2 population at 26, 27, and 28 generations. All parameters were estimated considering all the ten independent replicates*.

a *LD was calculated between adjacent SNPs from QMSim*.

b *SNP_after_, overlapping markers segregating in all three populations*.

### Genetic Connectedness Between Populations

#### Principal Component Analysis (PCA)

In order to better assess the population composition of the animals and to graphically display the results, we performed a PCA by decomposition of the genomic relationship matrix (G). Principal components were assessed using the flag “–*pca”* of PLINK 2.0 (Chang et al., 2015).

#### Consistency of Gametic Phase

The consistency of gametic phase was defined by the Pearson correlation of signed LD (measured by r) values between two populations [Line1 vs. Line2; Line1 vs. F1 (F1-1, F1-2, F1-3, and F1-4); Line2 vs. F1 (F1-1, F1-2, F1-3, and F1-4)]. The LD level between two SNP markers was measured by r^2^, in which r^2^ = $\frac{D^{2}}{f\left(A\right)f\left(a\right)f\left(B\right)f\left(b\right)}$; where *D* = *f*(*AB*)−*f*(*A*)*f*(*B*), and *f*(*AB*), *f*(*A*), *f*(*a*), *f*(*B*), and *f*(*b*) are observed frequencies of haplotype AB and alleles A, a, B, and b, respectively (Hill and Robertson, 1968). The LD levels were obtained by the flag “*–r2 dprime”* using the PLINK 2.0 software (Chang et al., 2015). The signed *r* value was obtained by taking the square root of the *r*^2^ value and assigning the appropriate sign based on the *D* value. Data was sorted into bins based on pair-wise SNP marker distance to determine the breakdown in the consistency of gametic phase across distances, and to assess the consistency of gametic phase at the smallest distances in the current panel, given the number of genotyped SNPs. For each distance bin, the signed *r* values were correlated between all pairs of populations using the *cor* basic function of the R statistical software (R Core Team, 2019).

#### Allele A Frequency Correlation

Assessment of the allele A frequency correlation across populations was based on the Pearson correlation. The allele frequency was calculated for each population individually using the option “–*freq*” from PLINK 2.0 (Chang et al., 2015).

### Genomic Prediction of Breeding Values

#### Methodological Scenarios

Comparisons between the ssGBLUP and WssGBLUP methods were based on the predictive ability of the GEBVs of the F1 animals. In other words, we aimed to identify the best scenario where the selection of purebred animals would result in the greatest crossbred performance (indicated by the GEBVs of crossbred animals). A total of five alternative scenarios (SC) were investigated: (SC1) ssGBLUP based on a single-trait model considering both purebred and crossbred animals in the training population; (SC2) ssGBLUP based on a multiple-trait model considering phenotypes recorded on purebred and crossbred training animals as different traits; (SC3) WssGBLUP based on a single-trait model including both purebred and crossbred animal datasets in the training population (and information from the three populations to estimate the SNP weights—further described); (SC4) WssGBLUP based on a single-trait model considering only purebred animals in the training population (and only the information from crossbred animals to estimate the SNP weights); (SC5) WssGBLUP based on a single-trait model considering only purebred animals in the training population (and their information to estimate the SNP weights). The main goal of SC4 was to account for the crossbred allele frequencies during the **G** calculation, and SC5 was performed to evaluate the use of only purebred information to predict crossbred performance.

The animals included in the training populations were purebred animals from generations six, seven, and eight (Line1), and generations 26, 27, and 28 (Line2). When crossbred animals were included in the training population, animals from F1-1 and F1-2 populations were used. The scenarios used to create the different training populations are summarized in Table 2. F1-3 and F1-4 were used as two different validation populations in all scenarios, in order to assess the impact of the genetic distance between training and validation populations in the genomic predictions.

**Table 2:** Structure of scenarios (SC) using the single-step Genomic Best Linear Unbiased Prediction (ssGBLUP) or weighted ssGBLUP (WssGBLUP) approaches, in terms of training population and single nucleotide polymorphism (SNP) weights.

**^a^Scenario**	**Training population**	**^b^SNP weights**	**Statistical model**
SC1	Purebred + Crossbred	^c^N/A	Single-trait model
SC2	Purebred + Crossbred	N/A	Multiple-trait model
SC3	Purebred + Crossbred	Purebred + Crossbred	Single-trait model
SC4	Purebred	Crossbred	Single-trait model
SC5	Purebred	Purebred	Single-trait model

a *SC1, ssGBLUP using a single-trait model and the training population composed of purebred and crossbred animals; SC2, ssGBLUP using a multiple-trait model and the training population composed of purebred and crossbred animals; SC3, WssGBLUP using a single-trait model, and training population and SNP weights based on both purebred and crossbred animals; SC4, WssGBLUP using a single-trait model, and training population composed only of purebred animals, and weights estimated from crossbred animals; and SC5, WssGBLUP using a single-trait model, and training population and SNP weights based only on purebred animals*.

b *Population used to estimate the SNP weights in the WssGBLUP*.

c *N/A, not applicable*.

#### ssGBLUP and WssGBLUP

The ssGBLUP and WssGBLUP methods were used to combine phenotypic, pedigree, and genotypic information. Therefore, the inverse of the **H** matrix (Misztal et al., 2009; Aguilar et al., 2010; Christensen and Lund, 2010) used in this study was created as:

(1) $$\begin{array}{l} {\text{H}}^{-1}={\text{A}}^{-1}+\;\left[\begin{array}{cc} 0 & 0\\ 0 & \tau {\left(0.95\text{G}+0.05{\text{A}}_{22}\right)}^{-1}-\omega {\text{A}}_{22}^{-1} \end{array}\right] \end{array}$$

Where **A** is the pedigree-based relationship matrix, which included up to five generations of animals with phenotypes or genotypes, **A**_22_ is the subset of the **A** matrix related to genotyped animals, the τ and ω values will be described further, and **G** is the genomic relationship matrix, which was created as follows (VanRaden, 2008):

(2) $$\begin{array}{l} \text{G}=\;\frac{\text{Z}\;\text{D}\;{\text{Z}}^{\prime }\;}{k},\text{with }\text{Z}=(\text{M}-\text{P}) \end{array}$$

Where **D** is a diagonal matrix with weights, *k* is a scale parameter defined as $2\sum_{j=1}^{n}p_{j}\left(1-p_{j}\right)$, **M** is a matrix of n SNPs for each animal, and **P** is a matrix containing two times the allele frequency of the second allele *p* at locus j (*p*_*j*_). In the ssGBLUP analyses, the **D** matrix was assumed as an identity matrix. In the WssGBLUP analyses, **D** was a diagonal matrix with values given by weights derived from the SNP solutions, as described by Wang et al. (2012). The SNP weights were obtained by back solving the GEBVs using the software BLUPF90 (Strandén and Garrick, 2009; Wang et al., 2012). First of all, the ssGBLUP was performed by using **D** matrix as an identity matrix (**I**). Then, the SNP weights were derived based on Strandén and Garrick (2009) and Wang et al. (2012):

(3) $$\begin{array}{l} \hat{u}=\lambda DM^{\prime }G^{-1}(GEBVs) \end{array}$$

Where û is a vector of estimated SNP effects, λ is the ratio of SNP variance to genetic variance, and GEBVs are the genomic estimated breeding values. The SNP weights to be considered in the next iteration (second iteration) were derived from the SNP effects as SNP variances:

(4) $$\begin{array}{l} d_{j}={û}_{j}^{2}2p_{j}\left(1-p_{j}\right) \end{array}$$

Where *d*_*j*_ is the *j* SNP weight (equivalent to *j* SNP variance); û is a vector of estimated *j* SNP effect; and *p* is the allele frequency of *j* SNP.

Consequently, a total of two iterations (i.e., using the identity matrix plus one iteration using the **D** matrix derived from SNP solutions) were used in the WssGBLUP because the second iteration provided higher GEBV accuracies in the preliminary analysis (Table S2). The SNP solutions were estimated using the POSTGSF90 software (Aguilar et al., 2014).

As genomic datasets were simulated, all individuals included in the pedigree also had genotypes. In order to make **G**^−1^ and **A**_**22**_^−1^ matrices compatible (Misztal et al., 2017; Oliveira et al., 2019), different values for the τ (from 0.9 to 2.5; defined at every 0.1) and ω (from 0.5 to 1.2; defined at every 0.1) parameters were tested. These ranges were chosen based on the literature (Misztal et al., 2017; Oliveira et al., 2019). As **G**^−1^ and **A**_**22**_^−1^ matrices were basically the same in all scenarios (i.e., the **A**_**22**_^−1^ matrix was the same in all scenarios, and **G**^−1^ matrix was the same in SC1, SC2, and SC3; and training crossbred animals were excluded from SC4 and SC5, but the validation crossbred animals remained on all SCs), τ and ω parameters were only tested using SC1. Thereafter, the tuning parameters that increased the accuracy and reduced the prediction bias of GEBVs were used in all analyses. Details about the methods used to calculate the accuracy and bias (based on regression coefficient) of GEBVs are described in section accuracy and regression coefficient. The inbreeding coefficient was estimated using the BLUPF90 family software (Misztal et al., 2002).

#### Statistical Models

The ssGBLUP and WssGBLUP analyses were performed using the BLUPF90 software (Misztal et al., 2002), based on single- and multiple-trait models. The single-trait models used in SC1, SC3, SC4, and SC5 are described as:

(5) $$\begin{array}{l} \text{y}=\text{Xb}+\text{Zu}+\text{e} \end{array}$$

Where **y**, **b**, **u** and **e** are the vectors of observations; fixed effects (mean, sex, and population); additive genetic random effects, **u** ~ N(**0**,${\;\sigma }_{u}^{2}\text{H}$); and random residuals, **e** ~ N(**0**,$\sigma_{e}^{2}\text{I}$), respectively. **X** and **Z** are the incidence matrices for **b** and **u**, respectively. $\sigma_{u}^{2}$ and $\sigma_{e}^{2}$ are the additive genetic and residual variances, respectively. Variance components were independently estimated for each scenario using the AIREMLF90 software (Misztal et al., 2002) and the **A** matrix, since it has been currently recommended in several ssGBLUP and WssGBLUP studies (Ali et al., 2019; Oliveira et al., 2019; Pocrnic et al., 2019). The multiple-trait model used in SC2 can be described as:

(6) $$\begin{array}{l} y_{3}=X_{3}b_{3}+Z_{3}u_{3}+e_{3} \end{array}$$

Where **y**_**3**_ is a vector of observations considering records from Line1, Line2, and F1 as three different traits; **b**_**3**_, **u**_**3**_, and **e**_**3**_ are the vectors of fixed effects (mean and sex), additive genetic random effects, **u**_**3**_ **~** N(**0**,**G**_**0**_ ⊗ **H**), and, random residuals, **e**_**3**_ **~** N(**0,R** ⊗ **I**), respectively. **X**_**3**_ and **Z**_**3**_ are the incidence matrices for the fixed and additive genetic effects, respectively. **G**_**0**_ and **R** are the additive genetic and residual variance-covariance matrices, respectively, described as:

(7) $$\begin{array}{l} {\text{G}}_{0}\text{=}\left[\begin{array}{ccc} \sigma_{{\text{u}}_{\text{Line}1}}^{2} & \sigma_{{\text{u}}_{\text{Line}1},{\text{u}}_{\text{Line}2}} & \sigma_{{\text{u}}_{\text{Line}1},{\text{u}}_{\text{F}1}}\\ \sigma_{{\text{u}}_{\text{Line}2},{\text{u}}_{\text{Line}1}} & \sigma_{{\text{u}}_{\text{Line}2}}^{2} & \sigma_{{\text{u}}_{\text{Line}2},{\text{u}}_{\text{F}1}}\\ \sigma_{{\text{u}}_{\text{F}1},{\text{u}}_{\text{Line}1}} & \sigma_{{\text{u}}_{\text{F}1},{\text{u}}_{\text{Line}2}} & \sigma_{{\text{u}}_{\text{F}1}}^{2} \end{array}\right] \end{array}$$

(8) $$\begin{array}{l} \text{R}=\left[\begin{array}{ccc} \sigma_{{\text{e}}_{\text{Line}1}}^{2} & 0 & 0\\ 0 & \sigma_{{\text{e}}_{\text{Line}2}}^{2} & 0\\ 0 & 0 & \sigma_{{\text{e}}_{\text{F}1}}^{2} \end{array}\right] \end{array}$$

Where $\sigma_{u_{Line1}}^{2}$, $\sigma_{u_{Line2}}^{2}$, and $\sigma_{u_{F1}}^{2}$ are the additive genetic variances for Line1, Line2, and F1, respectively; σ_*u*_ is the additive genetic (co)variance between pairs of populations; $\sigma_{e_{Line1}}^{2}$, $\sigma_{e_{Line2}}^{2}$, and $\sigma_{e_{F1}}^{2}$ are the residual variances for Line1, Line2, and F1, respectively.

### Accuracy and Regression Coefficient

The predictive ability of tested scenarios was evaluated based on a comparison of GEBVs and True Breeding Values (TBVs) of F1 populations. The main goal of the current study was to evaluate the predictive performance of genomic models when purebred parents are selected to produce crossbred progeny with higher genetic breeding value and improved performance, both indicated by higher GEBVs. Therefore, accuracies of genomic predictions were estimated as the Pearson correlation coefficients calculated between GEBVs and TBVs, for the validation populations (F1-3 and F1-4). In addition, the regression coefficient (an indicator of inflation or deflation of the TBVs on GEBVs) was assessed using a linear regression model of TBVs on GEBVs, for the validation animals. Paired Student's *t* test (Rosner, 1982) was applied to verify significant differences (*P* < 0.05) between accuracies and the regression coefficient from different scheme pairs by using the *t-test* function available in the R software (R Core Team, 2019).

## Results

### Variance and Covariance Components

Genetic parameters and (co)variance components estimated in the different simulated scenarios using the **A** matrix are shown in Table 3. In general, variance components estimated from SIM1, SIM2, SIM3, and SIM5 ranged from 0.03 to 0.05 for the additive genetic variance, and from 0.08 to 0.09 for the residual variance. Heritability estimated in SIM1, SIM2, SIM3, and SIM5 ranged from 0.26 to 0.40, which were consistent with the initial value used in the simulation process (*h*^2^ equal to 0.33). For the Line2 and F1 populations in the SIM4, additive genetic variance and *h*^2^ were underestimated (additive genetic variance equal to 0.01, and *h*^2^ ranged from 0.11 to 0.13) in comparison to the other scenarios. Genetic correlations across populations in the different scenarios ranged from moderate to high (from 0.71 to 0.99).

**Table 3:** Mean and standard deviation (in parentheses) of variance and covariance components and genetic parameters estimated for Line1, Line2, and F1 populations.

	**Line1**			**Line2**			**F1^1^**			**r_Line1,Line2_**	**r_Line1,F1_**	**r_Line2,F1_**
	**$\sigma_{u}^{2}$**	**$\sigma_{e}^{2}$**	***h*^2^**	**$\sigma_{u}^{2}$**	**$\sigma_{e}^{2}$**	***h*^2^**	**$\sigma_{u}^{2}$**	**$\sigma_{e}^{2}$**	***h*^2^**			
SIM1	0.05 (0.008)	0.08 (0.006)	0.40 (0.044)	0.05 (0.005)	0.09 (0.003)	0.36 (0.027)	0.04 (0.005)	0.09 (0.004)	0.33 (0.035)	0.71 (0.183)	0.81 (0.127)	0.95 (0.044)
SIM2	0.05 (0.004)	0.08 (0.004)	0.39 (0.026)	0.04 (0.005)	0.09 (0.002)	0.31 (0.030)	0.04 (0.005)	0.09 (0.004)	0.28 (0.034)	0.83 (0.147)	0.87 (0.125)	0.98 (0.019)
SIM3	0.05 (0.004)	0.09 (0.004)	0.36 (0.030)	0.05 (0.004)	0.08 (0.003)	0.36 (0.026)	0.04 (0.006)	0.09 (0.004)	0.32 (0.036)	0.83 (0.141)	0.91 (0.085)	0.95 (0.054)
SIM4	0.04 (0.005)	0.09 (0.004)	0.34 (0.032)	0.01 (0.004)	0.09 (0.002)	0.11 (0.037)	0.01 (0.003)	0.10 (0.001)	0.13 (0.026)	0.96 (0.089)	0.99 (0.009)	0.96 (0.085)
SIM5	0.05 (0.005)	0.08 (0.004)	0.38 (0.031)	0.03 (0.006)	0.09 (0.003)	0.27 (0.043)	0.03 (0.006)	0.09 (0.005)	0.26 (0.044)	0.74 (0.132)	0.86 (0.098)	0.94 (0.057)

*SIM1, simulated dataset with heritability explained by the quantitative trait loci (h^2^_QTL_) = 0; SIM2, simulated dataset with h^2^_QTL_ = 0.11 and 198 QTLs; SIM3, simulated dataset with h^2^_QTL_ = 0.11 and 4,500 QTLs; SIM4, simulated dataset with h^2^_QTL_ = 0.33 and 198 QTLs; SIM5, simulated dataset with h^2^_QTL_ = 0.33 and 4,500 QTLs; $\sigma_{u}^{2}$, additive genetic variance; $\sigma_{e}^{2}$, residual variance; h^2^, heritability; r_Line1,Line2_, genetic correlation between Line1 and Line2; r_Line1,F1_, genetic correlation between Line1 and F1; r_Line2,F1_, genetic correlation between Line2 and F1*.

1 *F1, F1-1, and F1-2 populations*.

### Genetic Connectedness Between Populations

#### Principal Component Analysis

**Figure 2:**
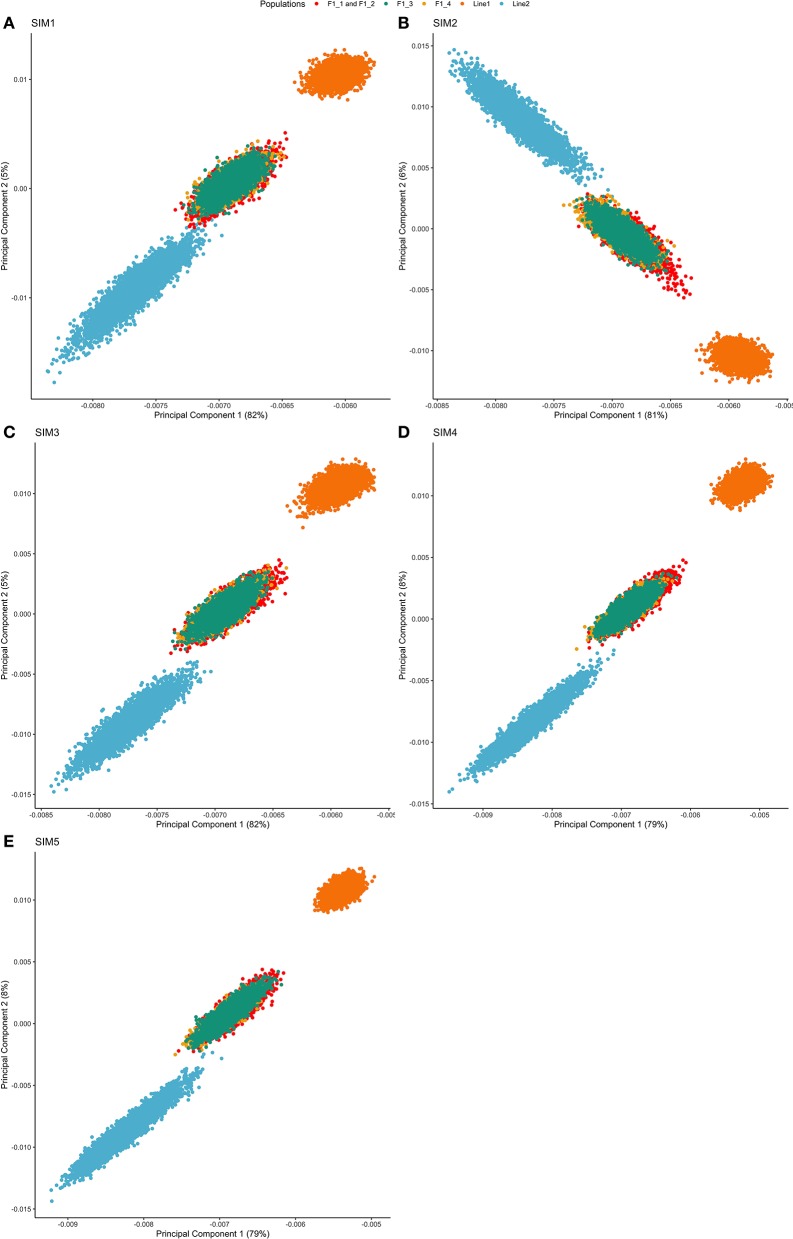
Principal component decomposition of the genomic relationship matrix of repetition 1 colored by breed-group. Letters represent the simulated scenarios: **(A)** Simulated scenario with heritability explained by the quantitative trait loci ($h_{QTL}^{2}$) equal to zero (SIM1); **(B)** $h_{QTL}^{2}$ equal to 1/3 of trait heritability (*h*^2^) (i.e., $h_{QTL}^{2}\;$equal to 0.11), and the number of QTLs equal to 198 (SIM2); **(C)** $h_{QTL}^{2}$ equal to 0.11 and the number of QTLs equal to 4,500 (SIM3); **(D)** $h_{QTL}^{2}$ equal to trait *h*^2^ (0.33), and the number of QTLs equal to 198 (SIM4); and **(E)** $h_{QTL}^{2}$ equal to 0.33 and the number of QTLs equal to 4,500 (SIM5).

Both purebred and F1 populations clustered separately, and the F1 animals clustered between both purebreds (as expected). This is shown by the first and second principal components (PC) of the genomic relationship matrix, in which the first principal component explained from 79 to 82% of the total variation (Figure 2). There was no projection overlapping in all five simulated scenarios, indicating that the populations were genetically divergent based on the relationship calculated from segregating SNPs.

#### Consistency of Gametic Phase

**Figure 3:**
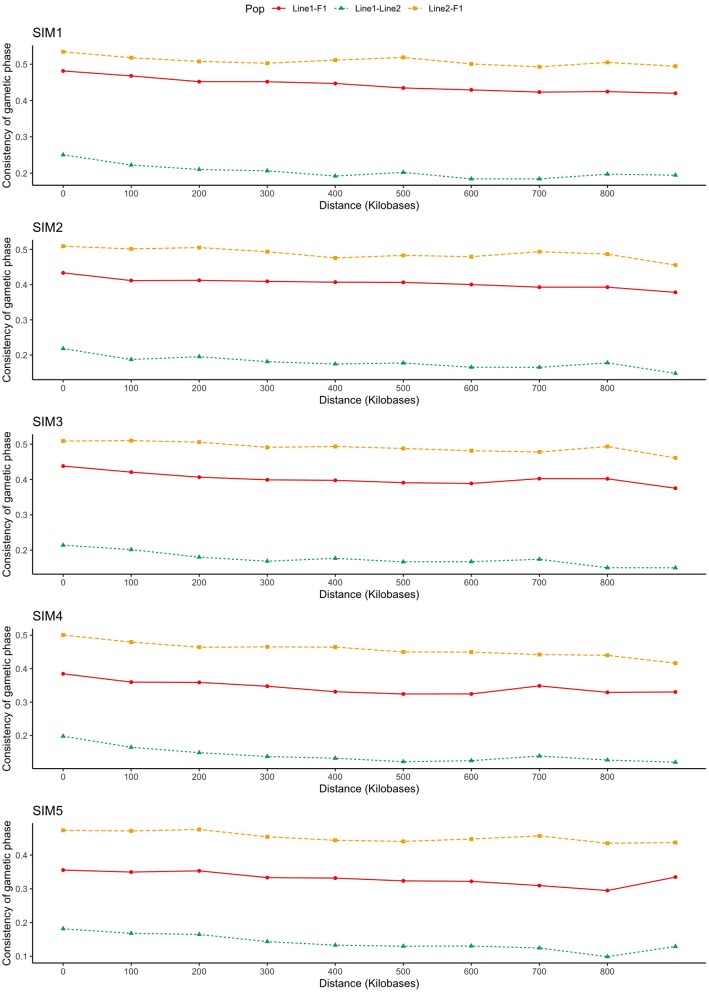
Consistency of gametic phase (Pearson correlations of signed *r* values) at given distances for three population pairs. SIM1: simulated scenario with heritability explained by the quantitative trait loci ($h_{QTL}^{2}$) equal to zero; SIM2: $h_{QTL}^{2}$ equal to 1/3 of trait heritability (*h*^2^) (i.e., $h_{QTL}^{2}\;$equal to 0.11), and the number of QTLs equal to 198; SIM3: $h_{QTL}^{2}$ equal to 0.11 and the number of QTLs equal to 4,500; SIM4: $h_{QTL}^{2}$ equal to trait *h*^2^ (0.33), and the number of QTLs equal to 198; and SIM5: $h_{QTL}^{2}$ equal to 0.33 and the number of QTLs equal to 4,500.

As presented in Figure 3, the consistency of gametic phase was reasonably low within purebred lines and low-to-moderate between purebred and crossbred individuals, even at the smallest SNP distance bins (from 0 to 60 kb). The consistency of gametic phase of SNP pairs separated by distances of up to 60 kb between Line1 and Line2 ranged from 0.13 (SIM4) to 0.22 (SIM1).

### Scaling Factors Used to Combine G^−1^ and ${\text{A}}_{22}^{-1}$ Matrices

**Figure 4:**
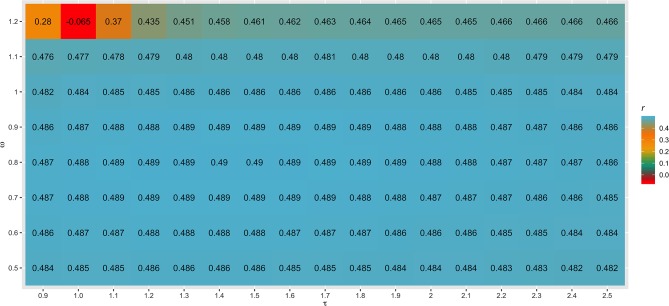
Heatmap of accuracy (*r*) for all combinations of τ and ω scaling factors to blend G^−1^ and ${\text{A}}_{22}^{-1}$ matrices when building the H matrix, using the dataset from the simulated scenario with heritability explained by the quantitative trait loci ($h_{QTL}^{2}$) equal to the trait heritability (*h*^2^) *of* 0.33 and 4,500 QTLs.

**Figure 5:**
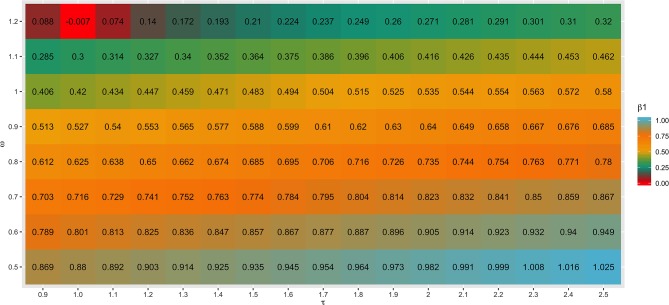
Heatmap of regression coefficient (β_1_) for all combinations of τ and ω scaling factors to blend G^−1^ and ${\text{A}}_{22}^{-1}$ matrices when building the H matrix, using the dataset from the simulated scenario with heritability explained by the quantitative trait loci ($h_{QTL}^{2}$) equal to the trait heritability (*h*^2^) *of* 0.33 and 4,500 QTLs.

Different values for τ (from 0.9 to 2.5) and ω (from 0.5 to 1.2) parameters were tested in SC1 when combining the **G**^−1^ and **A**_**22**_^−1^ matrices. Changes in accuracies and regression coefficients when using these different values are shown in Figures 4 and 5, respectively. In summary, small or no variation in the validation accuracies were observed when comparing different values of τ and ω (Figure 4), except for the combination of low τ and high ω that resulted in the lowest accuracies. This might be explained by an inappropriate combination of tuning parameters. However, a great impact of τ and ω combination was observed in the regression coefficients (Figure 5). Among all tested values, the combination of τ equal to 2.2 and ω equal to 0.5 yielded the least biased GEBVs (i.e., the regression coefficient was closer to one). Consequently, those τ and ω values were used in further analyses for all scenarios evaluated.

**Figure 6:**
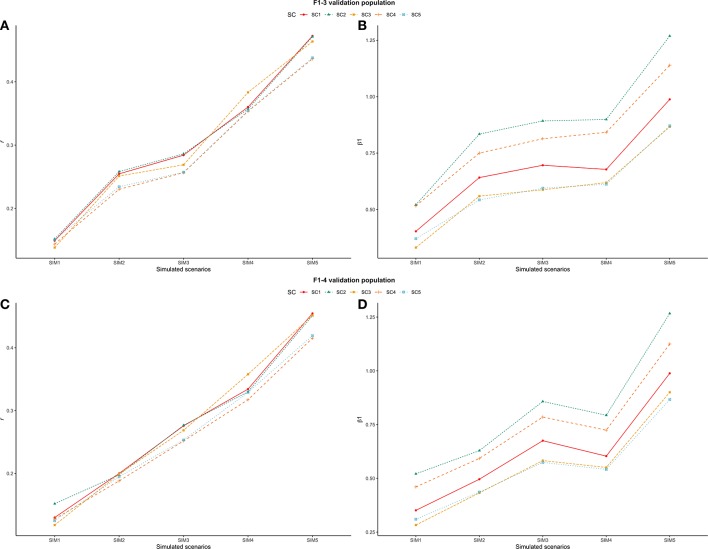
Trend line for average validation accuracy (*r*, **A,C**) and regression coefficient (β_1_, **B,D**) across all scenarios: ssGBLUP based on a single-trait model considering both purebred and crossbred animals in the training population (SC1); ssGBLUP based on a multiple-trait model to consider phenotypes recorded on purebred and crossbred training animals as different traits (SC2); WssGBLUP based on a single-trait model considering both purebred and crossbred animals in the training population (and information from both populations to estimate the SNP weights (SC3); WssGBLUP based on a single-trait model considering only purebred animals in the training population (and only the information from crossbred animals to estimate the SNP weights) (SC4); and WssGBLUP based on a single-trait model considering only purebred animals in the training population (and their information to estimate the SNP weights) (SC5); and simulated scenarios: heritability explained by the quantitative trait loci (h^2^_QTL_) equal to zero (SIM1); $h_{QTL}^{2}$ equal to 1/3 of trait heritability (*h*^2^) (i.e., $h_{QTL}^{2}\;$equal to 0.11), and the number of QTLs equal to 198 (SIM2); $h_{QTL}^{2}$ equal to 0.11 and the number of QTLs equal to 4,500 (SIM3); $h_{QTL}^{2}$ equal to trait *h*^2^ (0.33), and the number of QTLs equal to 198 (SIM4); and $h_{QTL}^{2}$ equal to 0.33 and the number of QTLs equal to 4,500 (SIM5). **(A,B)** represent F1-3 validation population and **(C,D)** represent F1-4 validation population.

With regards to the different simulated scenarios, when only a fraction (or nothing) of the trait *h*^2^ was attributed to the QTL effects $\left(h_{QTL}^{2}\right)$, most combinations of τ and ω parameters yielded less accurate and highly biased GEBVs (validation accuracies were low and the regression coefficients were far from one). This suggests that the genetic architecture of the trait has a great effect on the performance of genomic predictions (Daetwyler et al., 2010). In this context, when the number of QTLs was high (4,500) and the *h*^2^ explained by them was equal to 0.33 (i.e., $h_{QTL}^{2}$ equal to the trait *h*^2^), greater validation accuracies were observed (Figures 6A,C) and the GEBV bias decreased (Figures 6B,D).

### Genomic Predictions

Due to a large number of scenarios investigated, the Results section will be split according to the validation population (F1-3 or F1-4).

#### F1-3 Validation Population

**Figure 7:**
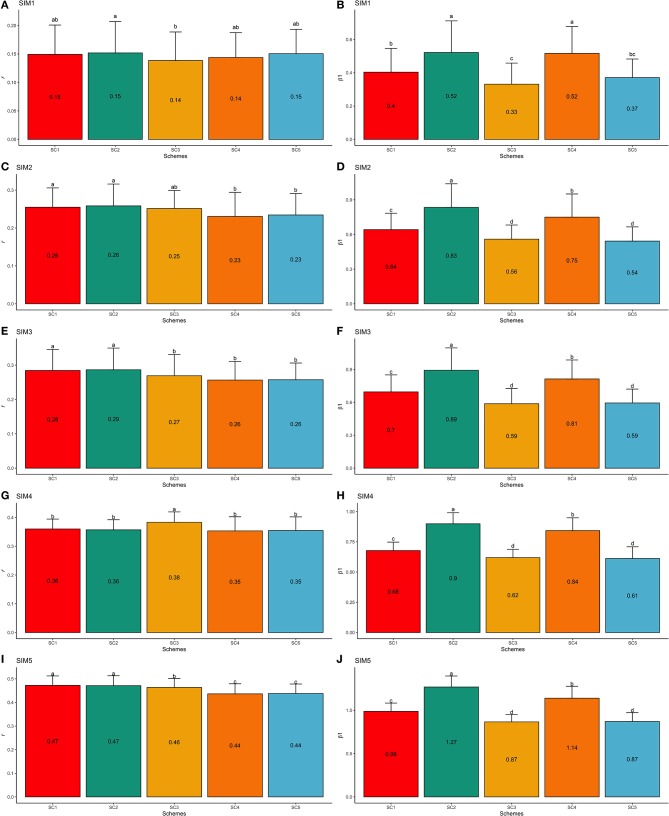
Average validation accuracies (*r* − **A,C,E,G,I**) and regression coefficients (β_1_− **B,D,F,H,J**) with, respectively standard deviations and different letters for each scenario representing significant differences (*P* < 0.05) for F1-3 validation population: ssGBLUP based on a single-trait model considering both purebred and crossbred animals in the training population (SC1); ssGBLUP based on a multiple-trait model to consider phenotypes recorded on purebred and crossbred training animals as different traits (SC2); WssGBLUP based on a single-trait model considering both purebred and crossbred animals in the training population (and information from both populations to estimate the SNP weights) (SC3); WssGBLUP based on a single-trait model considering only purebred animals in the training population (and only the information from crossbred animals to estimate the SNP weights) (SC4); and WssGBLUP based on a single-trait model considering only purebred animals in the training population (and their information to estimate the SNP weights) (SC5). Simulated scenarios: heritability explained by the quantitative trait loci ($h_{QTL}^{2}$) equal to zero (SIM1); $h_{QTL}^{2}$ equal to 1/3 of trait heritability (*h*^2^) (i.e., $h_{QTL}^{2}\;$equal to 0.11), and the number of QTLs equal to 198 (SIM2); $h_{QTL}^{2}$ equal to 0.11 and the number of QTLs equal to 4,500 (SIM3); $h_{QTL}^{2}$ equal to trait *h*^2^ (0.33), and the number of QTLs equal to 198 (SIM4); and $h_{QTL}^{2}$ equal to 0.33 and the number of QTLs equal to 4,500 (SIM5).

SIM1 is the simulation scenario that yielded the lowest GEBV accuracy and the highest bias estimates (e.g., regression coefficient far from one). The average GEBV accuracies in SIM1 ranged from 0.14 (SC3 and SC4) to 0.15 (SC1, SC2, and SC5; Figure 7A), and the regression coefficients ranged from 0.33 (SC3) to 0.52 (SC2 and SC4; Figure 7B). On the other hand, the simulated scenario with the highest accuracy and lowest bias (e.g., regression coefficient close one) was the SIM5. In SIM5, the average GEBV accuracies ranged from 0.44 (SC4 and SC5) to 0.47 (SC1 and SC2; Figure 7I), and the regression coefficients ranged from 0.87 (SC3 and SC5) to 1.27 (SC2; Figure 7J).

#### F1-4 Validation Population

**Figure 8:**
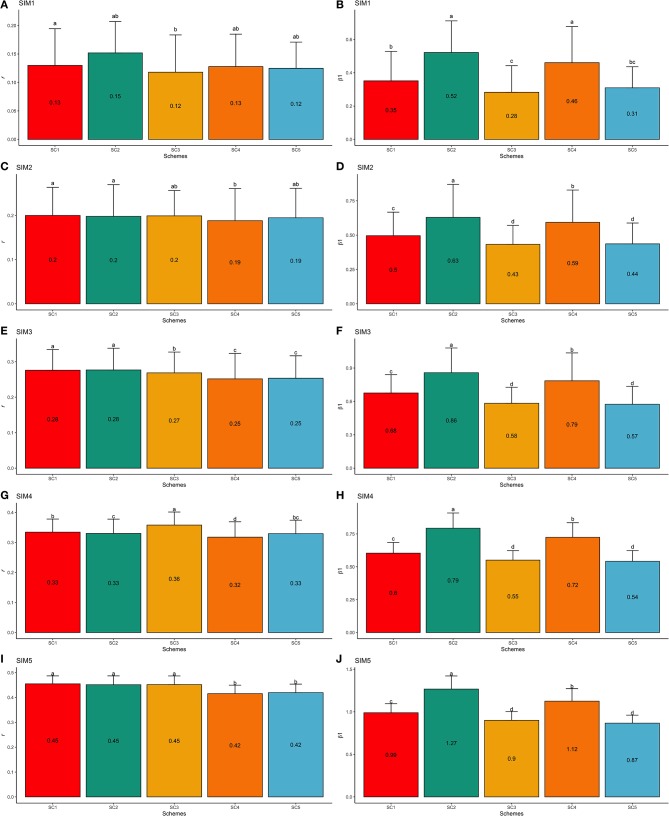
Average validation accuracies (*r* − **A,C,E,G,I**) and regression coefficients (β_1_− **B,D,F,H,J**) with, respectively standard deviations and different letters for each scenario representing significant differences (*P* < 0.05) for F1-4 validation population: ssGBLUP based on a single-trait model considering both purebred and crossbred animals in the training population (SC1); ssGBLUP based on a multiple-trait model to consider phenotypes recorded on purebred and crossbred training animals as different traits (SC2); WssGBLUP based on a single-trait model considering both purebred and crossbred animals in the training population (and information from both populations to estimate the SNP weights) (SC3); WssGBLUP based on a single-trait model considering only purebred animals in the training population (and only the information from crossbred animals to estimate the SNP weights) (SC4); and WssGBLUP based on a single-trait model considering only purebred animals in the training population (and their information to estimate the SNP weights) (SC5). Simulated scenarios: heritability explained by the quantitative trait loci ($h_{QTL}^{2}$) equal to zero (SIM1); $h_{QTL}^{2}$ equal to 1/3 of trait heritability (*h*^2^) (i.e., $h_{QTL}^{2}\;$equal to 0.11), and the number of QTLs equal to 198 (SIM2); $h_{QTL}^{2}$ equal to 0.11 and the number of QTLs equal to 4,500 (SIM3); $h_{QTL}^{2}$ equal to trait *h*^2^ (0.33), and the number of QTLs equal to 198 (SIM4); and $h_{QTL}^{2}$ equal to 0.33 and the number of QTLs equal to 4,500 (SIM5).

Similarly to the F1-3 validation set, the simulated scenarios SIM1 and SIM5 yielded the lowest and highest predictive abilities, respectively. Using the F1-4 validation population (one generation farther from the F1-3 training population) from the SIM1 dataset, the GEBV validation accuracy reduced by 13.98% when compared to the F1-3 validation set. Thus, the GEBV accuracies ranged from 0.12 (SC3 and SC5; SIM1) to 0.15 (SC2; SIM1; Figure 8A), and regression coefficients ranged from 0.28 (SC3; SIM1) to 0.52 (SC2; SIM1; Figure 8B). Based on the F1-4 validation set from SIM5, the validation accuracy reduced by 3.86% compared to F1-3. The accuracies ranged from 0.42 (SC4 and SC5; SIM5) to 0.46 (SC1, SC2, and SC3; SIM5; Figure 8I), and the regression coefficients ranged from 0.87 (SC5; SIM5) to 1.27 (SC2; SIM5; Figure 8J).

The GEBV accuracies and regression coefficients for the other simulated scenarios (SIM2–SIM4) are presented in Figures 7C–G, 8C–G for F1-3 and F1-4 validation populations, respectively. Furthermore, the GEBV accuracies and regression coefficients calculated for each replicate are shown in Tables S3, S4 for F1-3 and F1-4 validation populations, respectively.

## Discussion

### Variance and Covariance Components

Genetic correlations for the simulated trait across populations in the different scenarios ranged from moderate-to-high, which indicates that Line1, Line2, and F1 are moderate-to-high genetically correlated. Núñez-Dominguez et al. (1993) reported a moderate-to-high genetic correlation between purebred-crossbred populations (ranging from 0.55 to 0.97) for live weight measurements (e.g., birth, weaning, and yearling weights). Additionally, Newman et al. (2002) also reported moderate-to-high estimates ranging from 0.48 to 1.00 for moderate-to-high heritability traits (e.g., carcass weight and percentage of intramuscular fat). Based on a literature review, Wientjes and Calus (2017) reported an average genetic correlation between purebred-crossbred pigs equal to 0.63, with 50% of the estimates between 0.45 and 0.87 (Wientjes and Calus, 2017). The majority of the correlations observed in the current study are at the high end of this range. Assuming the exclusively moderate-to-high genetic relationship between all population pairs and a large training population, genomic predictions between those populations are expected to be reasonably accurate (Daetwyler et al., 2015).

### Genetic Connectedness Between Populations

Principal Components Analysis absorbs the information of allele frequencies into a reduced number of independent variables, facilitating the interpretation of potential population structure. The first two PCs showed a clear separation between populations Line1 and Line2, and the F1 animals clustered between both purebred lines (Figure 2). Additionally, despite the differences in the F1 generations (F1-1, F1-2, F1-3, and F1-4), all of them were grouped in a single cluster.

The first principal component (PC1) was strongly correlated with Line1 in all simulation scenarios, except for SIM2 (Figure 2). This fact highlights that PC1 increases with an increasing relationship in Line1. However, different results can be expected due to the stochastic nature of the simulation analysis and the sampling process to create the training population (as observed for SIM2). Thus, the general pattern of PC1 in comparison to Line1 can be seen as a genomic index that ensures the strong relationship among individuals belonging to the same line.

The improvement of the predictive ability of two distinct training and validation populations (e.g., purebred and crossbred) depends on the similarity or consistency of gametic phase between the SNPs and QTLs across populations. By increasing the relationship distance between individuals, the genomic distance in which the linkage phase will be consistent across populations decreases. As presented in Figure 3, the consistency of gametic phase was reasonably low to moderate among all populations' pairs. As expected, Line1 and Line2 presented the lowest consistency of gametic phase. Populations paired with F1 (i.e., Line1 vs. F1, and Line2 vs. F1) presented the highest consistency of gametic phase.

Both results, PCA and consistency of gametic phase, suggest that better accuracies of genomic predictions could be attained when using a single-training population as the SNP effects seem to be population-specific. In other words, the lower predictive ability could be expected when SNP effects estimated based on Line1 is applied to Line2, or across any combination presented. However, those assumptions are contrasted by the genetic correlation between Line1 and Line2 (i.e., moderate-to-high genetic correlations).

Even though a moderate-to-high genetic correlation was observed between Line1 and Line2, there was still population stratification. The contrasting results from both analyses (genetic correlation vs. PCA + consistency of gametic phase + allele frequency correlation) might be explained by: (i) the similar selection direction for all populations (i.e., selection of lower EBV animals from Line1, Line2, and F1), which could result in a high genetic correlation across these populations for the trait under selection; (ii) single-trait selection, in which only the alleles associated with the trait (or in high LD) would contribute to higher genetic correlation between the populations, but not all the markers spread across the genome; and, (iii) specific population parameters (e.g., LD, effective population size, different number of generations, and SNP marker segregation). In other words, when simulating a genomic dataset, one needs to specify: (1) the number of QTLs affecting the trait (this can be interpreted as the causal mutations affecting the trait, which are usually the same across populations), and (2) the number of markers in the dataset, in which some will be in LD with the QTLs simulated, while the others might be non-related to the trait and spread out across the whole genome. Thus, it is not surprising that the QTL effects (causal mutations) and their allele frequencies across populations (Line1 and Line2) for the trait under study were similar, which is realistic.

### Scaling Factors Used to Combine G^−1^ and ${\text{A}}_{22}^{-1}$ Matrices

The ssGBLUP and WssGBLUP methods assume that the statistical model is correct and that allelic frequencies come from the base population (Oliveira et al., 2019). However, these assumptions usually do not hold in practice, which can result in prediction bias (Vitezica et al., 2011). In this context, **G**^−1^ and **A**_**22**_^−1^ matrices are usually not on the same scale (Misztal et al., 2017; Oliveira et al., 2019). In order to obtain better prediction accuracies and reduce the bias, Tsuruta et al. (2011) and Misztal et al. (2013) reported that scaling factors should be used when combining **G**^−1^ and **A**_**22**_^−1^ matrices to create the **H** matrix.

The different scaling factors tested in this study had no or small influence in the validation accuracies (Figure 4). These findings are in agreement with those reported by Oliveira et al. (2019), who also observed a small impact of these parameters in the reliability of genomic predictions using real datasets from three Canadian dairy cattle breeds (Holstein, Jersey, and Ayrshire). On the other hand, Koivula et al. (2018) reported significant differences in the validation reliabilities across few pairwise combinations of τ and ω parameters.

As initially reported by Tsuruta et al. (2011) and Misztal et al. (2013), different combinations of τ and ω also had a great impact on the bias estimates in the current study (Figure 5). This can be explained by the reduction in the variance of the predicted genetic values resulting in larger regression coefficients (Martini et al., 2018), depending on the scaling factor combination used. In general, changes in τ had a smaller impact on the bias than changes in ω, as also reported by Oliveira et al. (2019). The best ω parameter assumed in this study (0.50) was lower than 1.00, which increases the importance of pedigree information on GEBV prediction. This is related to the fact that this study used a simulated dataset and therefore, the pedigree is complete and precise.

### Genomic Prediction of Breeding Values

#### Accuracies

In general, significant differences were observed across scenarios (Figures 7, 8 for F1-3 and F1-4, respectively).

##### Single-trait vs. multiple-trait model

In general, single- and multiple-trait models yielded similar results across all the simulated scenarios and validation populations (Figures 7, 8). Calus et al. (2014) reported that a single-trait model can result in similar predictive accuracies compared to multiple-trait or non-linear models when assuming a high genetic correlation between the populations analyzed together. On the other hand, greater predictive ability was observed by using multiple-trait or non-linear models when the populations were less genetically correlated (Calus et al., 2014). Therefore, the genetic connectedness between populations in a pooled-breed analysis might interfere with the model performance (Calus et al., 2014). In the present study, all population pairs presented moderate-to-high genetic correlations for the trait simulated (Table 3), which might explain the similar predictive ability across all the scenarios investigated.

##### ssGBLUP vs. WssGBLUP

For the SIM1, SIM2, SIM3, and SIM5, SC1, and SC2 (using the ssGBLUP method) yielded the highest GEBV accuracies. This suggests that the ssGBLUP method, using either a single- or multiple-trait model, performs better than WssGBLUP for polygenic traits in crossbred animals. We expected that WssGBLUP would perform better for the scenarios SIM2 through SIM5, and especially for SIM4 and SIM5. Lourenco et al. (2017) reported that for less polygenic traits (such as the simulated scenarios mentioned above), the accuracy might be higher when using WssGBLUP instead of ssGBLUP. WssGBLUP is advantageous for traits with a reduced number of causative genes because its assumption is similar to the genetic architecture of those traits: a finite number of markers affecting the trait. However, no pattern was observed across those simulated scenarios for WssGBLUP. In SIM4, the SC3 scheme (characterized by the WssGBLUP using purebred and crossbred populations to estimate the SNP weights and predict the GEBVs) yielded the highest accuracy. The genetic variation of the trait in SIM4 is completely controlled by few QTLs. In other words, SIM4 is a less polygenic scenario across all others.

Accounting for breed-specific allele frequencies could potentially increase the predictive ability in multi-breed models (Dekkers, 2007; Ibánêz-Escriche et al., 2009; Christensen et al., 2014). This can be accounted for through WssGBLUP (e.g., Sevillano et al., 2019). However, small differences were observed by using ssGBLUP and WssGBLUP in the present study. The similarity across scenarios might also be partially explained by the data simulation structure that resulted in a moderate-to-high genetic correlation across all population pairs, as they were all selected based on a single trait. Additionally, the allele A frequency correlations among all population pairs ranged from moderate (0.24–0.48; Line1 vs. Line2; Tables S5A–S5E) to high (0.61–0.85; Line1 vs. F1, and Line2 vs. F1; Tables S5A–S5E). In real datasets, differences in allele frequencies diverge due to different breeding goals across generations and populations/breeds. Similarly, Lourenco et al. (2016) did not observe differences in GEBV accuracies when using breed-specific allele frequencies to build the **G** matrix in the genomic evaluation of crossbred animals. Furthermore, Ibánêz-Escriche et al. (2009) also reported that genomic selection for crossbred populations using models that fit the breed-specific effects of SNP alleles are not necessary.

Scenarios SC4 and SC5 had fewer individuals in the training population than SC1 and SC3 scenarios, which could lead to greater accuracies of both larger training population scenarios. Therefore, additional analyses using the same-size training populations of SC1 and SC3 vs. SC4 and SC5 were performed (Tables S6A–S6E). Small or no differences were observed by using a balanced dataset for SC1 and SC3 scenarios, which do not change the conclusions previously reported. Therefore, the differences between ssGBLUP and WssGBLUP were still small. However, the way the estimation of SNP weights has been carried out in this and other studies (Ibánêz-Escriche et al., 2009; Lourenco et al., 2016) might not be optimal. The weights derivation used is the easiest way to implement the WssGBLUP in commercial breeding programs, which justify the application of the method. Alternative ways to derive the SNP weights have been proposed and might result in better predictive ability (Su et al., 2014; Karaman et al., 2019), for example through Bayesian approaches.

##### Purebred vs. jointly purebred and crossbred training populations

There are studies indicating that the addition of crossbred information in the training population to predict crossbred performance has a positive impact on the predictive ability of GEBVs (Bijma and van Arendonk, 1998; Bijma et al., 2001; Lutaaya et al., 2002; Fragomeni et al., 2016; Iversen et al., 2017). However, Pocrnic et al. (2019), using a dataset with purebred and crossbred pigs, did not observe differences in GEBV accuracies when the SNP effects were estimated based solely on purebreds or obtained through combining purebred and crossbred animals in the training set. In this study, the high genetic correlations between purebred and crossbred populations (from 0.81 to 0.99 between Line1 and F1, and 0.94 to 0.98 between Line2 and F1) might explain the small differences observed when including crossbred information in the training population (from SC1 to SC4 vs. SC5, Figures 7, 8). In general, moderate-to-high genetic correlations between purebreds and crossbred populations tend to result in higher GEBV prediction accuracies (Pocrnic et al., 2019). This might be due to the purebred information's ability to capture most of the crossbred genetic variation when larger training sets are available.

#### Regression Coefficients

Significant differences were observed among regression coefficients estimated in the different scenarios (Figures 7, 8 for F1-3 and F1-4, respectively). The GEBV bias obtained in SC3 and SC5 may be due to the inefficient estimation of SNP weights in predicting crossbred information, as a merged dataset (purebred and crossbred) or just purebred information was used to estimate the SNP weights to predict GEBVs in the crossbred animals in SC3 and SC5, respectively. As previously mentioned, alternative ways to derive the SNP weights have been proposed, which could lead to better predictive performance (Su et al., 2014; Karaman et al., 2019). In general, less biased GEBVs were obtained in SC2, which is in agreement with several studies in the literature with regards to the superiority of multiple-trait models to predict the performance of crossbred populations (Tusell et al., 2016; Pocrnic et al., 2019).

#### Comparing Simulated Datasets

In general, higher GEBV accuracies and regression coefficients close to one were obtained for SIM4 and SIM5 (simulated datasets in which all genetic variances were explained by the QTLs). Simulated scenarios with a small or null number of QTLs (SIM1, SIM2, and SIM3) might lead to higher GEBV accuracy when using Bayesian variable selection models (Habier et al., 2011). In composite beef cattle populations, the accuracy of GEBVs averaged over twenty economically important traits ranged from 0.38 to 0.40 across different scenarios (Piccoli et al., 2017). It is worth noting that as the $h_{QTL}^{2}$ reduced, the GEBV accuracy decreased and the bias increased. This indicates that simulated scenarios with $h_{QTL}^{2}\;$lower than *h*^2^ (total heritability) have a greater bias due to the fact that the relationship matrix does not account for an infinite number of loci (Kennedy et al., 1988).

As previously reported by Calus et al. (2014), a greater predictive performance of the multiple-trait model was observed under the lower relationship between purebred-crossbred populations (SIM1) than in a simulated higher relationship scenario (SIM4) (Figures 7, 8 and Table 3). In general, the crossbred information included in the training population during the GEBV estimation process had a greater impact on GEBV accuracy while using a simulated scenario with lower genetic correlation between purebred-crossbred populations, than other simulated scenarios with higher genetic correlation between populations.

#### F1-3 vs. F1-4 Validation Populations

Smaller differences in accuracies and regression coefficients were observed when the F1-3 validation population was used in comparison to the F1-4. This might be related to the smaller genetic gap between training and the F1-3 validation population (Muir, 2007; Goddard, 2009).

## Conclusions

In general, the ssGBLUP method based on a single-trait model considering both purebred and crossbred (F1) animals in the training population (SC1), and ssGBLUP based on a multiple-trait model considering phenotypes recorded on purebred and crossbred training animals as different traits (SC2), yielded the highest accuracies and lowest biases of GEBVs. Considering the current stratification of the genotyped population [low consistency of gametic phase across purebred and F1 populations; clear distinction of populations based on PCA; but moderate-to-high genetic correlations (ranging from 0.71 to 0.99)] for the simulated trait across populations, the ssGBLUP using a single-trait model and a purebred and crossbred (F1) training population's scenario (SC1) is recommended. The SC1 resulted in a similar performance of genomic evaluations in crossbred animals and it is reasonably easy to be implemented in practical situations. Further studies using real datasets should be performed to validate these findings.

## Data Availability Statement

The raw data supporting the conclusions of this article will be made available by the authors, without undue reservation, to any qualified researcher. Requests to access the datasets should be directed to Amanda B. Alvarenga (alvarena@purdue.edu) or Luiz F. Brito (britol@purdue.edu).

## Author Contributions

AA carried out all the analyses, and wrote the original draft. AA, RV, HO, FS, and LB conceived and designed this study. AA, HO, FS, RV, and LB interpreted and discussed the results. AA, RV, HO, FS, DM, PL, and LB reviewed and approved the final manuscript.

### Conflict of Interest

The authors declare that the research was conducted in the absence of any commercial or financial relationships that could be construed as a potential conflict of interest. The reviewer MC declared a past co-authorship with one of the authors FS to the handling editor.
