# Supplementary material for: Comparing Alternative Single-Step GBLUP Approaches and Training Population Designs for Genomic Evaluation of Crossbred Animals
Source: Front Genet. 2020 Apr 9;11:263. doi: 10.3389/fgene.2020.00263 (PMC7162606; doi:10.3389/fgene.2020.00263)
Supplement: Supplementary file 1 [file Table_1.docx]

**Table S1A. SIM1- Descriptive analyses of each simulation replicate for the number of SNPs before and after genotype quality control, and mean (standard deviation) of inbreeding coefficient (F), phenotype, allele A frequency** $\boldsymbol{(}\boldsymbol{\rho}_{\boldsymbol{A}}\boldsymbol{)}$**, and linkage disequilibrium (LD) for Line1, Line2 and F1s (F1) populations.**

| **Rep** | **SNPs_b_** | **SNPs_a_** | **F** | | | **Phenotype** | | | $\boldsymbol{\rho}_{\boldsymbol{A}}$ | | | **LD** | |
| --- | --- | --- | --- | --- | --- | --- | --- | --- | --- | --- | --- | --- | --- |
|  |  |  | **Line1** | **Line2** | **F1** | **Line1** | **Line2** | **F1** | **Line1** | **Line2** | **F1** | **Line1** | **Line2** |
| 1 | 48,485 | 45,019 | 0.02 (0.008) | 0.12 (0.026) | 0.01 (0.010) | -0.70 (0.133) | -2.81 (0.127) | -1.80 (0.128) | 0.34 (0.107) | 0.30 (0.125) | 0.33 (0.109) | 0.20 | 0.29 |
| 2 | 48,301 | 44,821 | 0.02 (0.010) | 0.12 (0.024) | 0.02 (0.012) | -0.72 (0.137) | -2.68 (0.131) | -1.75 (0.134) | 0.33 (0.107) | 0.30 (0.125) | 0.33 (0.109) | 0.20 | 0.30 |
| 3 | 48,342 | 44,889 | 0.02 (0.009) | 0.13 (0.031) | 0.02 (0.011) | -0.76 (0.134) | -2.95 (0.129) | -1.91 (0.131) | 0.33 (0.107) | 0.30 (0.125) | 0.33 (0.110) | 0.20 | 0.30 |
| 4 | 48,174 | 44,723 | 0.02 (0.009) | 0.13 (0.025) | 0.02 (0.014) | -0.74 (0.134) | -2.78 (0.130) | -1.81 (0.131) | 0.33 (0.108) | 0.30 (0.125) | 0.33 (0.110) | 0.19 | 0.30 |
| 5 | 48,127 | 44,791 | 0.02 (0.011) | 0.12 (0.026) | 0.02 (0.012) | -0.75 (0.136) | -2.82 (0.137) | -1.83 (0.129) | 0.33 (0.108) | 0.30 (0.126) | 0.32 (0.110) | 0.19 | 0.29 |
| 6 | 48,339 | 44,510 | 0.02 (0.011) | 0.13 (0.024) | 0.02 (0.015) | -0.79 (0.137) | -2.85 (0.129) | -1.86 (0.129) | 0.34 (0.107) | 0.30 (0.126) | 0.33 (0.109) | 0.20 | 0.32 |
| 7 | 48,225 | 44,887 | 0.02 (0.011) | 0.12 (0.025) | 0.02 (0.013) | -0.76 (0.139) | -2.82(0.130) | -1.84 (0.136) | 0.33 (0.107) | 0.30 (0.125) | 0.33 (0.110) | 0.20 | 0.28 |
| 8 | 48,121 | 44,864 | 0.02 (0.010) | 0.12 (0.027) | 0.02 (0.011) | -0.73 (0.134) | -2.78 (0.126) | -1.81 (0.127) | 0.33 (0.108) | 0.30 (0.124) | 0.33 (0.109) | 0.19 | 0.30 |
| 9 | 48,303 | 45,014 | 0.02 (0.009) | 0.12 (0.026) | 0.02 (0.013) | -0.78 (0.138) | -2.86 (0.134) | -1.88 (0.125) | 0.33 (0.108) | 0.30 (0.124) | 0.33 (0.110) | 0.19 | 0.30 |
| 10 | 48,194 | 44,827 | 0.02 (0.009) | 0.13 (0.027) | 0.02 (0.014) | -0.74 (0.139) | -2.95 (0.135) | -1.92 (0.134) | 0.33 (0.108) | 0.30 (0.125) | 0.33 (0.110) | 0.19 | 0.31 |

SIM1: simulated dataset with heritability explained by the quantitative trait loci (h²_QTL_) = 0; Rep: simulation replicate; SNP_b_: number of single nucleotide polymorphisms before quality control; SNP_a_: number of single nucleotide polymorphisms that remained after quality control; F: inbreeding coefficient; LD: linkage disequilibrium between adjacent SNPs (r² metric) from QMSim; Line1: line1 at six, seven and eight generations; Line2: line2 at 26, 27 and 28 generations; F1: F1-1 and F1-2 crossbred populations at generation 1.

**Table S1B. SIM2- Descriptive analyses of each simulation replicate for the number of SNPs before and after genotype quality control, and mean (standard deviation) of inbreeding coefficient (F), phenotype, allele A frequency** $\boldsymbol{(}\boldsymbol{\rho}_{\boldsymbol{A}}\boldsymbol{)}$**, and linkage disequilibrium (LD) for Line1, Line2 and F1s (F1) populations.**

| **Rep** | **SNPs_b_** | **SNPs_a_** | **F** | | | **Phenotype** | | | $\boldsymbol{\rho}_{\boldsymbol{A}}$ | | | **LD** | |
| --- | --- | --- | --- | --- | --- | --- | --- | --- | --- | --- | --- | --- | --- |
|  |  |  | **Line1** | **Line2** | **F1** | **Line1** | **Line2** | **F1** | **Line1** | **Line2** | **F1** | **Line1** | **Line2** |
| 1 | 48,195 | 43,768 | 0.02 (0.009) | 0.14 (0.027) | 0.02 (0.015) | -0.72 (0.136) | -2.57 (0.121) | -1.69 (0.125) | 0.34 (0.107) | 0.29 (0.127) | 0.33 (0.109) | 0.19 | 0.31 |
| 2 | 48,312 | 44,530 | 0.02 (0.008) | 0.12 (0.026) | 0.02 (0.014) | -0.69 (0.134) | -2.72 (0.129) | -1.76 (0.126) | 0.34 (0.107) | 0.30 (0.125) | 0.33 (0.109) | 0.19 | 0.30 |
| 3 | 48,099 | 43,779 | 0.02 (0.008) | 0.14 (0.025) | 0.02 (0.016) | -0.68 (0.128) | -2.64 (0.124) | -1.72 (0.126) | 0.34 (0.107) | 0.29 (0.125) | 0.33 (0.109) | 0.18 | 0.31 |
| 4 | 48,331 | 43,263 | 0.02 (0.009) | 0.16 (0.025) | 0.02 (0.017) | -0.68 (0.135) | -2.67 (0.123) | -1.73 (0.121) | 0.34 (0.106) | 0.29 (0.128) | 0.33 (0.109) | 0.20 | 0.32 |
| 5 | 48,321 | 44,296 | 0.02 (0.011) | 0.12 (0.025) | 0.02 (0.013) | -0.78 (0.142) | -2.70 (0.124) | -1.78 (0.129) | 0.33 (0.107) | 0.30 (0.125) | 0.33 (0.109) | 0.19 | 0.30 |
| 6 | 48,219 | 43,267 | 0.02 (0.009) | 0.14 (0.027) | 0.02 (0.014) | -0.73 (0.132) | -2.77 (0.126) | -1.79 (0.127) | 0.34 (0.107) | 0.29 (0.128) | 0.33 (0.109) | 0.19 | 0.32 |
| 7 | 48,210 | 44,095 | 0.02 (0.009) | 0.14 (0.030) | 0.01 (0.016) | -0.72 (0.139) | -2.71 (0.126) | -1.79 (0.128) | 0.34 (0.107) | 0.30 (0.126) | 0.33 (0.110) | 0.19 | 0.31 |
| 8 | 48,297 | 44,848 | 0.02 (0.009) | 0.11 (0.026) | 0.01 (0.013) | -0.80 (0.135) | -2.85 (0.129) | -1.89 (0.137) | 0.33 (0.107) | 0.30 (0.124) | 0.33 (0.110) | 0.20 | 0.29 |
| 9 | 48,287 | 44,368 | 0.02 (0.010) | 0.12 (0.027) | 0.02 (0.013) | -0.67 (0.135) | -2.67 (0.122) | -1.74 (0.127) | 0.33 (0.107) | 0.30 (0.125) | 0.33 (0.110) | 0.18 | 0.30 |
| 10 | 48,146 | 43,733 | 0.02 (0.009) | 0.146 (0.026) | 0.02 (0.017) | -0.675 (0.132) | -2.64 (0.124) | -1.69 (0.127) | 0.34 (0.107) | 0.29 (0.127) | 0.33 (0.109) | 0.19 | 0.30 |

SIM2: simulated dataset with heritability explained by the quantitative trait loci (h²_QTL_) = 0.11 and 198 QTLs; Rep: simulation replicate; SNP_b_: number of single nucleotide polymorphisms before quality control; SNP_a_: number of single nucleotide polymorphisms that remained after quality control; F: inbreeding coefficient; LD: linkage disequilibrium between adjacent SNPs (r² metric) from QMSim; Line1: line1 at six, seven and eight generations; Line2: line2 at 26, 27 and 28 generations; F1: F1-1 and F1-2 crossbred populations at generation 1.

**Table S1C. SIM3- Descriptive analyses of each simulation replicate for the number of SNPs before and after genotype quality control, and mean (standard deviation) of inbreeding coefficient (F), phenotype, allele A frequency** $\boldsymbol{(}\boldsymbol{\rho}_{\boldsymbol{A}}\boldsymbol{)}$**, and linkage disequilibrium (LD) for Line1, Line2 and F1s (F1) populations.**

| **Rep** | **SNPs_b_** | **SNPs_a_** | **F** | | | **Phenotype** | | | $\boldsymbol{\rho}_{\boldsymbol{A}}$ | | | **LD** | |
| --- | --- | --- | --- | --- | --- | --- | --- | --- | --- | --- | --- | --- | --- |
|  |  |  | **Line1** | **Line2** | **F1** | **Line1** | **Line2** | **F1** | **Line1** | **Line2** | **F1** | **Line1** | **Line2** |
| 1 | 48,266 | 44,360 | 0.02 (0.009) | 0.12 (0.025) | 0.02 (0.012) | -0.76 (0.133) | -2.81 (0.135) | -1.83 (0.132) | 0.33 (0.108) | 0.29 (0.127) | 0.32 (0.111) | 0.19 | 0.30 |
| 2 | 48,202 | 44,416 | 0.02 (0.010) | 0.12 (0.024) | 0.02 (0.013) | -0.74 (0.136) | -2.86 (0.129) | -1.85 (0.133) | 0.33 (0.107) | 0.29 (0.125) | 0.33 (0.110) | 0.19 | 0.29 |
| 3 | 48,343 | 44,120 | 0.02 (0.009) | 0.13 (0.024) | 0.02 (0.014) | -0.68 (0.134) | -2.74 (0.125) | -1.75 (0.127) | 0.33 (0.107) | 0.29 (0.126) | 0.33 (0.110) | 0.19 | 0.33 |
| 4 | 48,234 | 43,980 | 0.02 (0.009) | 0.13 (0.025) | 0.02 (0.014) | -0.73 (0.138) | -2.82 (0.132) | -1.84 (0.129) | 0.33 (0.107) | 0.29 (0.127) | 0.33 (0.110) | 0.20 | 0.33 |
| 5 | 48,126 | 44,306 | 0.02 (0.009) | 0.11 (0.027) | 0.02 (0.013) | -0.71 (0.134) | -2.80 (0.134) | -1.79 (0.133) | 0.33 (0.108) | 0.30 (0.126) | 0.33 (0.110) | 0.18 | 0.31 |
| 6 | 48,309 | 42,081 | 0.02 (0.009) | 0.17 (0.025) | 0.02 (0.019) | -0.74 (0.139) | -2.92 (0.131) | -1.88 (0.131) | 0.34 (0.106) | 0.28 (0.129) | 0.33 (0.108) | 0.19 | 0.35 |
| 7 | 48,372 | 44,867 | 0.01 (0.009) | 0.12 (0.025) | 0.02 (0.014) | -0.74 (0.135) | -2.68 (0.13) | -1.78 (0.125) | 0.33 (0.107) | 0.30 (0.126) | 0.33 (0.110) | 0.20 | 0.30 |
| 8 | 48,374 | 44,784 | 0.01 (0.008) | 0.11 (0.026) | 0.02 (0.013) | -0.76 (0.136) | -2.70 (0.124) | -1.78 (0.134) | 0.33 (0.107) | 0.29 (0.126) | 0.33 (0.110) | 0.19 | 0.30 |
| 9 | 48,068 | 43,360 | 0.02 (0.009) | 0.15 (0.029) | 0.02 (0.016) | -0.74 (0.142) | -2.89 (0.135) | -1.89 (0.134) | 0.34 (0.106) | 0.29 (0.128) | 0.33 (0.109) | 0.19 | 0.32 |
| 10 | 48,314 | 44,699 | 0.02 (0.010) | 0.12 (0.025) | 0.02 (0.013) | -0.75 (0.136) | -2.73 (0.127) | -1.79 (0.127) | 0.33 (0.106) | 0.30 (0.126) | 0.33 (0.110) | 0.20 | 0.31 |

SIM3: simulated dataset with heritability explained by the quantitative trait loci (h²_QTL_) = 0.11 and 4,500 QTLs; Rep: simulation replicate; SNP_b_: single nucleotide polymorphism before quality control; SNP_a_: single nucleotide polymorphism remained after quality control; F: inbreeding coefficient; LD: linkage disequilibrium between adjacent SNPs (r² metric) from QMSim; Line1: line1 at six, seven and eight generations; Line2: line2 at 26, 27 and 28 generations; F1: F1-1 and F1-2 crossbred populations at generation 1.

**Table S1D. SIM4- Descriptive analyses of each simulation replicate for the number of SNPs before and after genotype quality control, and mean (standard deviation) of inbreeding coefficient (F), phenotype, allele A frequency** $\boldsymbol{(}\boldsymbol{\rho}_{\boldsymbol{A}}\boldsymbol{)}$**, and linkage disequilibrium (LD) for Line1, Line2 and F1s (F1) populations.**

| **Rep** | **SNPs_b_** | **SNPs_a_** | **F** | | | **Phenotype** | | | $\boldsymbol{\rho}_{\boldsymbol{A}}$ | | | **LD** | |
| --- | --- | --- | --- | --- | --- | --- | --- | --- | --- | --- | --- | --- | --- |
|  |  |  | **Line1** | **Line2** | **F1** | **Line1** | **Line2** | **F1** | **Line1** | **Line2** | **F1** | **Line1** | **Line2** |
| 1 | 48,170 | 40,437 | 0.02 (0.011) | 0.18 (0.029) | 0.02 (0.019) | -0.70 (0.127) | -1.98 (0.098) | -1.38 (0.108) | 0.34 (0.105) | 0.27 (0.130) | 0.32 (0.109) | 0.19 | 0.37 |
| 2 | 48,353 | 43,123 | 0.02 (0.008) | 0.14 (0.028) | 0.02 (0.013) | -0.71 (0.138) | -2.19 (0.100) | -1.49 (0.116) | 0.34 (0.106) | 0.29 (0.128) | 0.33 (0.110) | 0.19 | 0.33 |
| 3 | 48,339 | 41,553 | 0.02 (0.011) | 0.17 (0.028) | 0.02 (0.018) | -0.62 (0.127) | -2.01 (0.103) | -1.34 (0.114) | 0.34 (0.105) | 0.27 (0.131) | 0.33 (0.108) | 0.19 | 0.40 |
| 4 | 48,232 | 42,267 | 0.02 (0.012) | 0.14 (0.032) | 0.02 (0.015) | -0.71 (0.132) | -2.02 (0.096) | -1.39 (0.115) | 0.34 (0.107) | 0.29 (0.128) | 0.33 (0.109) | 0.18 | 0.33 |
| 5 | 48,237 | 42,291 | 0.02 (0.010) | 0.16 (0.033) | 0.01 (0.014) | -0.65 (0.125) | -1.74 (0.094) | -1.23 (0.106) | 0.34 (0.106) | 0.28 (0.129) | 0.33 (0.109) | 0.19 | 0.33 |
| 6 | 48,364 | 42,964 | 0.02 (0.012) | 0.14 (0.029) | 0.02 (0.014) | -0.69 (0.130) | -1.87 (0.102) | -1.32 (0.109) | 0.34 (0.106) | 0.29 (0.127) | 0.33 (0.108) | 0.18 | 0.40 |
| 7 | 48,158 | 42,824 | 0.02 (0.010) | 0.13 (0.027) | 0.02 (0.013) | -0.69 (0.123) | -1.96 (0.101) | -1.36 (0.113) | 0.33 (0.108) | 0.29 (0.128) | 0.32 (0.109) | 0.19 | 0.33 |
| 8 | 48,212 | 42,115 | 0.02 (0.009) | 0.17 (0.029) | 0.02 (0.020) | -0.71 (0.128) | -1.92 (0.099) | -1.33 (0.107) | 0.34 (0.106) | 0.29 (0.128) | 0.33 (0.108) | 0.18 | 0.35 |
| 9 | 48,261 | 41,754 | 0.02 (0.010) | 0.15 (0.037) | 0.02 (0.017) | -0.72 (0.130) | -2.04 (0.097) | -1.42 (0.110) | 0.34 (0.105) | 0.28 (0.129) | 0.33 (0.108) | 0.19 | 0.35 |
| 10 | 48,176 | 42,091 | 0.02 (0.010) | 0.16 (0.028) | 0.02 (0.017) | -0.70 (0.133) | -2.08 (0.100) | -1.43 (0.115) | 0.34 (0.106) | 0.28 (0.128) | 0.33 (0.108) | 0.18 | 0.43 |

SIM4: simulated dataset with heritability explained by the quantitative trait loci (h²_QTL_) = 0.33 and 198 QTLs; Rep: simulation replicate; SNP_b_: number of single nucleotide polymorphisms before quality control; SNP_a_: number of single nucleotide polymorphisms that remained after quality control; F: inbreeding coefficient; LD: linkage disequilibrium between adjacent SNPs (r² metric) from QMSim; Line1: line1 at six, seven and eight generations; Line2: line2 at 26, 27 and 28 generations; F1: F1-1 and F1-2 crossbred populations at generation 1.

**Table S1E. SIM5- Descriptive analyses of each simulation replicate for the number of SNPs before and after genotype quality control, and mean (standard deviation) of inbreeding coefficient (F), phenotype, allele A frequency** $\boldsymbol{(}\boldsymbol{\rho}_{\boldsymbol{A}}\boldsymbol{)}$**, and linkage disequilibrium (LD) for Line1, Line2 and F1s (F1) populations.**

| **Rep** | **SNPs_b_** | **SNPs_a_** | **F** | | | **Phenotype** | | | $\boldsymbol{\rho}_{\boldsymbol{A}}$ | | | **LD** | |
| --- | --- | --- | --- | --- | --- | --- | --- | --- | --- | --- | --- | --- | --- |
|  |  |  | **Line1** | **Line2** | **F1** | **Line1** | **Line2** | **F1** | **Line1** | **Line2** | **F1** | **Line1** | **Line2** |
| 1 | 48,111 | 41,643 | 0.02 (0.008) | 0.15 (0.029) | 0.01 (0.015) | -0.74 (0.132) | -2.78 (0.117) | -1.79 (0.123) | 0.34 (0.105) | 0.27 (0.130) | 0.33 (0.109) | 0.19 | 0.35 |
| 2 | 48,236 | 42,088 | 0.02 (0.010) | 0.15 (0.028) | 0.02 (0.014) | -0.75 (0.135) | -2.66 (0.111) | -1.77 (0.121) | 0.34 (0.105) | 0.28 (0.129) | 0.33 (0.109) | 0.18 | 0.37 |
| 3 | 48,218 | 41,320 | 0.02 (0.008) | 0.16 (0.028) | 0.02 (0.017) | -0.73 (0.141) | -2.80 (0.124) | -1.81 (0.132) | 0.34 (0.104) | 0.27 (0.131) | 0.32 (0.108) | 0.18 | 0.36 |
| 4 | 48,209 | 43,390 | 0.02 (0.009) | 0.12 (0.026) | 0.01 (0.013) | -0.68 (0.140) | -2.68 (0.125) | -1.72 (0.127) | 0.34 (0.107) | 0.29 (0.126) | 0.33 (0.109) | 0.19 | 0.34 |
| 5 | 48,235 | 43,272 | 0.02 (0.008) | 0.12 (0.023) | 0.02 (0.013) | -0.81 (0.141) | -2.76 (0.118) | -1.84 (0.128) | 0.34 (0.107) | 0.29 (0.128) | 0.32 (0.110) | 0.19 | 0.33 |
| 6 | 48,263 | 42,686 | 0.02 (0.009) | 0.14 (0.028) | 0.02 (0.015) | -0.81 (0.137) | -2.85 (0.131) | -1.89 (0.127) | 0.34 (0.106) | 0.28 (0.131) | 0.32 (0.110) | 0.18 | 0.38 |
| 7 | 48,257 | 42,392 | 0.02 (0.009) | 0.13 (0.025) | 0.01 (0.012) | -0.67 (0.139) | -2.66 (0.129) | -1.71 (0.129) | 0.34 (0.106) | 0.28 (0.129) | 0.32 (0.110) | 0.20 | 0.35 |
| 8 | 48,278 | 42,554 | 0.02 (0.009) | 0.13 (0.028) | 0.02 (0.014) | -0.76 (0.135) | -2.64 (0.117) | -1.76 (0.124) | 0.34 (0.106) | 0.28 (0.128) | 0.32 (0.110) | 0.19 | 0.39 |
| 9 | 48,282 | 42,033 | 0.02 (0.008) | 0.16 (0.028) | 0.02 (0.016) | -0.74 (0.132) | -2.55 (0.116) | -1.68 (0.125) | 0.34 (0.106) | 0.28 (0.130) | 0.33 (0.109) | 0.19 | 0.39 |
| 10 | 48,200 | 42,719 | 0.02 (0.010) | 0.14 (0.029) | 0.02 (0.014) | -0.69 (0.130) | -2.58 (0.113) | -1.68 (0.127) | 0.34 (0.106) | 0.28 (0.129) | 0.32 (0.109) | 0.18 | 0.31 |

SIM5: simulated dataset with heritability explained by the quantitative trait loci (h²_QTL_) = 0.33 and 4,500 QTLs; Rep: simulation replicate; SNP_before_: number of single nucleotide polymorphisms before quality control; SNP_after_: single nucleotide polymorphisms that remained after quality control; F: inbreeding coefficient; LD: linkage disequilibrium between adjacent SNPs (r² metric) from QMSim; Line1: line1 at six, seven and eight generations; Line2: line2 at 26, 27 and 28 generations; F1: F1-1 and F1-2 populations of crossbreed at generation 1.
