# Supplementary material for: Comparing Alternative Single-Step GBLUP Approaches and Training Population Designs for Genomic Evaluation of Crossbred Animals
Source: Front Genet. 2020 Apr 9;11:263. doi: 10.3389/fgene.2020.00263 (PMC7162606; doi:10.3389/fgene.2020.00263)
Supplement: Supplementary file 2 [file Table_2.docx]

**Table S2. Accuracy (***r***) and regression coefficient (**$\boldsymbol{\beta}_{\boldsymbol{1}}$**) of true breeding value (TBV) on genomic estimated breeding value (GEBV) for simulated population SIM1 and SIM5 (simulation replicate 1) via weighted ssGBLUP using jointly purebred and crossbreed dataset for iterations two to five.**

| Iterations | SIM1 | | SIM5 | |
| --- | --- | --- | --- | --- |
|  | *r* | $\beta_{1}$ | *r* | $\beta_{1}$ |
| 2 | 0.16 | 0.36 | 0.51 | 1.00 |
| 3 | 0.15 | 0.29 | 0.49 | 0.87 |
| 4 | 0.15 | 0.27 | 0.47 | 0.83 |
| 5 | 0.15 | 0.27 | 0.46 | 0.80 |

SIM1: simulated dataset with heritability explained by the quantitative trait loci (h²_QTL_) = 0; SIM5: simulated dataset with h²_QTL_ = 0.33 and 4,500 QTLs; *r*: accuracy represented by Pearson correlation between GEBV and TBV; and β_1_: regression coefficient of a regression model of TBV on GEBV.
