# Supplementary material for: Comparing Alternative Single-Step GBLUP Approaches and Training Population Designs for Genomic Evaluation of Crossbred Animals
Source: Front Genet. 2020 Apr 9;11:263. doi: 10.3389/fgene.2020.00263 (PMC7162606; doi:10.3389/fgene.2020.00263)
Supplement: Supplementary file 4 [file Table_4.docx]

**Table S4A. SIM1 Accuracy (***r***) and regression coefficient (**$\boldsymbol{\beta}_{\boldsymbol{1}}$**) of true breeding value (TBV) on genomic estimated breeding value (GEBV) for the different genetic methodology schemes tested using the F1-4 validation group.**

| **Rep** | **SC1** | | **SC2_L1** | | **SC2_L2** | | **SC2** | | **SC3** | | **SC4** | | **SC5** | |
| --- | --- | --- | --- | --- | --- | --- | --- | --- | --- | --- | --- | --- | --- | --- |
|  | *r* | $\boldsymbol{\beta}_{\boldsymbol{1}}$ | *r* | $\boldsymbol{\beta}_{\boldsymbol{1}}$ | *r* | $\boldsymbol{\beta}_{\boldsymbol{1}}$ | *r* | $\boldsymbol{\beta}_{\boldsymbol{1}}$ | *r* | $\boldsymbol{\beta}_{\boldsymbol{1}}$ | *r* | $\boldsymbol{\beta}_{\boldsymbol{1}}$ | *r* | $\boldsymbol{\beta}_{\boldsymbol{1}}$ |
| 1 | 0.08 | 0.23 | 0.08 | 0.23 | 0.08 | 0.25 | 0.17 | 0.54 | 0.07 | 0.17 | 0.08 | 0.27 | 0.09 | 0.23 |
| 2 | 0.10 | 0.28 | 0.10 | 0.27 | 0.11 | 0.35 | 0.08 | 0.31 | 0.09 | 0.20 | 0.10 | 0.37 | 0.08 | 0.20 |
| 3 | 0.05 | 0.14 | 0.06 | 0.16 | 0.06 | 0.18 | 0.13 | 0.42 | 0.04 | 0.10 | 0.06 | 0.19 | 0.07 | 0.17 |
| 4 | 0.07 | 0.19 | 0.07 | 0.19 | 0.08 | 0.24 | 0.25 | 0.81 | 0.07 | 0.16 | 0.10 | 0.34 | 0.12 | 0.27 |
| 5 | 0.10 | 0.27 | 0.12 | 0.32 | 0.10 | 0.33 | 0.06 | 0.23 | 0.09 | 0.22 | 0.10 | 0.35 | 0.11 | 0.27 |
| 6 | 0.21 | 0.58 | 0.19 | 0.54 | 0.23 | 0.74 | 0.14 | 0.49 | 0.20 | 0.49 | 0.22 | 0.86 | 0.21 | 0.58 |
| 7 | 0.20 | 0.53 | 0.19 | 0.52 | 0.20 | 0.63 | 0.16 | 0.54 | 0.20 | 0.47 | 0.19 | 0.66 | 0.16 | 0.39 |
| 8 | 0.12 | 0.37 | 0.11 | 0.34 | 0.13 | 0.47 | 0.20 | 0.80 | 0.10 | 0.27 | 0.10 | 0.46 | 0.09 | 0.29 |
| 9 | 0.24 | 0.66 | 0.20 | 0.57 | 0.25 | 0.79 | 0.18 | 0.64 | 0.23 | 0.55 | 0.21 | 0.73 | 0.18 | 0.44 |
| 10 | 0.12 | 0.27 | 0.12 | 0.28 | 0.12 | 0.33 | 0.14 | 0.43 | 0.09 | 0.20 | 0.12 | 0.37 | 0.12 | 0.26 |

SIM1: simulated dataset with heritability explained by the quantitative trait loci (h²_QTL_) = 0; Rep: simulation replicate; SC1: single-trait ssGBLUP with a training population represented by purebred and crossbred animals; SC2-L1: multi-trait ssGBLUP with a training population represented by purebred and crossbred animals, and estimation of F1s performance based on results from Line1; SC2-L2: multi-trait ssGBLUP with a training population represented by purebred and crossbred animals, and estimation of F1s performance based on results from Line2; SC3: single-trait WssGBLUP with a training population represented by purebred and crossbred animals; SC4: single-trait WssGBLUP with a training population represented by purebreds and weights estimated from information of crossbred animals; SC5: single-trait WssGBLUP with a training population represented by purebreds; *r*: accuracy represented by Pearson’s correlation between GEBV and TBV; and β_1_: regression coefficient of a regression model of TBV on GEBV.

**Table S4B. SIM2 Accuracy (***r***) and regression coefficient (**$\boldsymbol{\beta}_{\boldsymbol{1}}$**) of true breeding value (TBV) on genomic estimated breeding value (GEBV) for the different genetic methodology schemes tested using the F1-4 validation group.**

| **Rep** | **SC1** | | **SC2_L1** | | **SC2_L2** | | **SC2** | | **SC3** | | **SC4** | | **SC5** | |
| --- | --- | --- | --- | --- | --- | --- | --- | --- | --- | --- | --- | --- | --- | --- |
|  | *r* | $\boldsymbol{\beta}_{\boldsymbol{1}}$ | *r* | $\boldsymbol{\beta}_{\boldsymbol{1}}$ | *r* | $\boldsymbol{\beta}_{\boldsymbol{1}}$ | *r* | $\boldsymbol{\beta}_{\boldsymbol{1}}$ | *r* | $\boldsymbol{\beta}_{\boldsymbol{1}}$ | *r* | $\boldsymbol{\beta}_{\boldsymbol{1}}$ | *r* | $\boldsymbol{\beta}_{\boldsymbol{1}}$ |
| 1 | 0.34 | 0.88 | 0.31 | 0.84 | 0.36 | 1.06 | 0.35 | 1.15 | 0.32 | 0.74 | 0.34 | 1.08 | 0.34 | 0.76 |
| 2 | 0.21 | 0.46 | 0.21 | 0.48 | 0.21 | 0.52 | 0.21 | 0.57 | 0.21 | 0.40 | 0.21 | 0.54 | 0.20 | 0.39 |
| 3 | 0.14 | 0.33 | 0.16 | 0.42 | 0.12 | 0.35 | 0.12 | 0.39 | 0.14 | 0.30 | 0.09 | 0.27 | 0.12 | 0.28 |
| 4 | 0.14 | 0.33 | 0.13 | 0.32 | 0.13 | 0.37 | 0.13 | 0.41 | 0.14 | 0.30 | 0.14 | 0.43 | 0.13 | 0.29 |
| 5 | 0.16 | 0.43 | 0.15 | 0.39 | 0.15 | 0.47 | 0.15 | 0.52 | 0.18 | 0.41 | 0.15 | 0.48 | 0.14 | 0.32 |
| 6 | 0.15 | 0.36 | 0.16 | 0.40 | 0.13 | 0.39 | 0.14 | 0.43 | 0.15 | 0.32 | 0.12 | 0.37 | 0.14 | 0.31 |
| 7 | 0.26 | 0.69 | 0.24 | 0.65 | 0.27 | 0.83 | 0.27 | 0.91 | 0.27 | 0.60 | 0.25 | 0.83 | 0.24 | 0.56 |
| 8 | 0.19 | 0.46 | 0.20 | 0.51 | 0.18 | 0.51 | 0.18 | 0.55 | 0.19 | 0.39 | 0.18 | 0.57 | 0.23 | 0.49 |
| 9 | 0.21 | 0.52 | 0.20 | 0.52 | 0.22 | 0.63 | 0.21 | 0.69 | 0.19 | 0.42 | 0.21 | 0.66 | 0.22 | 0.49 |
| 10 | 0.21 | 0.52 | 0.20 | 0.50 | 0.21 | 0.62 | 0.21 | 0.68 | 0.21 | 0.45 | 0.21 | 0.69 | 0.21 | 0.47 |

SIM2: simulated dataset with heritability explained by the quantitative trait loci (h²_QTL_) = 0.11 and 198 QTLs; Rep: simulation replicate ; SC1: single-trait ssGBLUP with a training population represented by purebred and crossbred animals; SC2-L1: multi-trait ssGBLUP with a training population represented by purebred and crossbred animals, and estimation of F1s performance based on results from Line1; SC2-L2: multi-trait ssGBLUP with a training population represented by purebred and crossbred animals, and estimation of F1s performance based on results from Line2; SC3: single-trait WssGBLUP with a training population represented by purebred and crossbred animals; SC4: single-trait WssGBLUP with a training population represented by purebreds and weights estimated from information of crossbred animals; SC5: single-trait WssGBLUP with a training population represented by purebreds; *r*: accuracy represented by Pearson’s correlation between GEBV and TBV; and β_1_: regression coefficient of a regression model of TBV on GEBV.

**Table S4C. SIM3 Accuracy (***r***) and regression coefficient (**$\boldsymbol{\beta}_{\boldsymbol{1}}$**) of true breeding value (TBV) on genomic estimated breeding value (GEBV) for the different genetic methodology schemes tested using the F1-4 validation group.**

| **Rep** | **SC1** | | **SC2_L1** | | **SC2_L2** | | **SC2** | | **SC3** | | **SC4** | | **SC5** | |
| --- | --- | --- | --- | --- | --- | --- | --- | --- | --- | --- | --- | --- | --- | --- |
|  | *r* | $\boldsymbol{\beta}_{\boldsymbol{1}}$ | *r* | $\boldsymbol{\beta}_{\boldsymbol{1}}$ | *r* | $\boldsymbol{\beta}_{\boldsymbol{1}}$ | *r* | $\boldsymbol{\beta}_{\boldsymbol{1}}$ | *r* | $\boldsymbol{\beta}_{\boldsymbol{1}}$ | *r* | $\boldsymbol{\beta}_{\boldsymbol{1}}$ | *r* | $\boldsymbol{\beta}_{\boldsymbol{1}}$ |
| 1 | 0.31 | 0.82 | 0.27 | 0.75 | 0.32 | 1.01 | 0.32 | 1.08 | 0.30 | 0.69 | 0.29 | 1.00 | 0.31 | 0.76 |
| 2 | 0.32 | 0.74 | 0.30 | 0.77 | 0.31 | 0.83 | 0.31 | 0.91 | 0.30 | 0.63 | 0.28 | 0.82 | 0.26 | 0.58 |
| 3 | 0.36 | 0.98 | 0.34 | 0.96 | 0.37 | 1.18 | 0.37 | 1.27 | 0.36 | 0.85 | 0.37 | 1.26 | 0.35 | 0.85 |
| 4 | 0.28 | 0.66 | 0.26 | 0.64 | 0.29 | 0.81 | 0.29 | 0.88 | 0.27 | 0.57 | 0.27 | 0.82 | 0.27 | 0.59 |
| 5 | 0.26 | 0.64 | 0.25 | 0.65 | 0.26 | 0.73 | 0.26 | 0.79 | 0.26 | 0.54 | 0.26 | 0.81 | 0.25 | 0.57 |
| 6 | 0.27 | 0.66 | 0.24 | 0.64 | 0.26 | 0.74 | 0.26 | 0.81 | 0.27 | 0.59 | 0.22 | 0.67 | 0.23 | 0.52 |
| 7 | 0.35 | 0.80 | 0.33 | 0.81 | 0.35 | 0.92 | 0.35 | 1.01 | 0.34 | 0.70 | 0.33 | 0.97 | 0.33 | 0.70 |
| 8 | 0.19 | 0.51 | 0.17 | 0.48 | 0.19 | 0.60 | 0.19 | 0.65 | 0.18 | 0.43 | 0.15 | 0.51 | 0.17 | 0.42 |
| 9 | 0.22 | 0.47 | 0.24 | 0.53 | 0.21 | 0.51 | 0.21 | 0.56 | 0.22 | 0.43 | 0.20 | 0.54 | 0.21 | 0.43 |
| 10 | 0.20 | 0.47 | 0.19 | 0.46 | 0.20 | 0.56 | 0.20 | 0.61 | 0.19 | 0.39 | 0.16 | 0.45 | 0.16 | 0.33 |

SIM3: simulated dataset with heritability explained by the quantitative trait loci (h²_QTL_) = 0.11 and 4,500 QTLs; Rep: simulation replicate ; SC1: single-trait ssGBLUP with a training population represented by purebred and crossbred animals; SC2-L1: multi-trait ssGBLUP with a training population represented by purebred and crossbred animals, and estimation of F1s performance based on results from Line1; SC2-L2: multi-trait ssGBLUP with a training population represented by purebred and crossbred animals, and estimation of F1s performance based on results from Line2; SC3: single-trait WssGBLUP with a training population represented by purebred and crossbred animals; SC4: single-trait WssGBLUP with a training population represented by purebreds and weights estimated from information of crossbred animals; SC5: single-trait WssGBLUP with a training population represented by purebreds; *r*: accuracy represented by Pearson’s correlation between GEBV and TBV; and β_1_: regression coefficient of a regression model of TBV on GEBV.

**Table S4D. SIM4 Accuracy (***r***) and regression coefficient (**$\boldsymbol{\beta}_{\boldsymbol{1}}$**) of true breeding value (TBV) on genomic estimated breeding value (GEBV) for the different genetic methodology schemes tested using the F1-4 validation group.**

| **Rep** | **SC1** | | **SC2_L1** | | **SC2_L2** | | **SC2** | | **SC3** | | **SC4** | | **SC5** | |
| --- | --- | --- | --- | --- | --- | --- | --- | --- | --- | --- | --- | --- | --- | --- |
|  | *r* | $\boldsymbol{\beta}_{\boldsymbol{1}}$ | *r* | $\boldsymbol{\beta}_{\boldsymbol{1}}$ | *r* | $\boldsymbol{\beta}_{\boldsymbol{1}}$ | *r* | $\boldsymbol{\beta}_{\boldsymbol{1}}$ | *r* | $\boldsymbol{\beta}_{\boldsymbol{1}}$ | *r* | $\boldsymbol{\beta}_{\boldsymbol{1}}$ | *r* | $\boldsymbol{\beta}_{\boldsymbol{1}}$ |
| 1 | 0.31 | 0.51 | 0.30 | 0.49 | 0.30 | 0.61 | 0.30 | -1.48 | 0.31 | 0.46 | 0.28 | 0.60 | 0.28 | 0.43 |
| 2 | 0.33 | 0.65 | 0.32 | 0.60 | 0.32 | 0.79 | 0.32 | -1.58 | 0.35 | 0.59 | 0.31 | 0.71 | 0.33 | 0.58 |
| 3 | 0.38 | 0.66 | 0.37 | 0.65 | 0.37 | 0.78 | 0.37 | -1.42 | 0.41 | 0.62 | 0.37 | 0.80 | 0.38 | 0.61 |
| 4 | 0.36 | 0.65 | 0.35 | 0.62 | 0.36 | 0.80 | 0.36 | -1.48 | 0.40 | 0.60 | 0.36 | 0.83 | 0.37 | 0.60 |
| 5 | 0.30 | 0.52 | 0.30 | 0.49 | 0.29 | 0.64 | 0.30 | -1.31 | 0.33 | 0.48 | 0.28 | 0.65 | 0.30 | 0.47 |
| 6 | 0.37 | 0.63 | 0.35 | 0.59 | 0.37 | 0.77 | 0.37 | -1.40 | 0.38 | 0.56 | 0.34 | 0.74 | 0.36 | 0.56 |
| 7 | 0.33 | 0.63 | 0.33 | 0.59 | 0.34 | 0.78 | 0.34 | -1.46 | 0.35 | 0.57 | 0.32 | 0.73 | 0.32 | 0.53 |
| 8 | 0.30 | 0.51 | 0.32 | 0.53 | 0.29 | 0.61 | 0.29 | -1.45 | 0.32 | 0.45 | 0.28 | 0.62 | 0.30 | 0.45 |
| 9 | 0.26 | 0.53 | 0.27 | 0.53 | 0.24 | 0.61 | 0.25 | -1.51 | 0.30 | 0.51 | 0.24 | 0.62 | 0.27 | 0.50 |
| 10 | 0.40 | 0.75 | 0.39 | 0.71 | 0.41 | 0.93 | 0.41 | -1.52 | 0.43 | 0.67 | 0.40 | 0.95 | 0.41 | 0.69 |

SIM4: simulated dataset with heritability explained by the quantitative trait loci (h²_QTL_) = 0.33 and 198 QTLs; Rep: simulation replicate ; SC1: single-trait ssGBLUP with a training population represented by purebred and crossbred animals; SC2-L1: multi-trait ssGBLUP with a training population represented by purebred and crossbred animals, and estimation of F1s performance based on results from Line1; SC2-L2: multi-trait ssGBLUP with a training population represented by purebred and crossbred animals, and estimation of F1s performance based on results from Line2; SC3: single-trait WssGBLUP with a training population represented by purebred and crossbred animals; SC4: single-trait WssGBLUP with a training population represented by purebreds and weights estimated from information of crossbred animals; SC5: single-trait WssGBLUP with a training population represented by purebreds; *r*: accuracy represented by Pearson’s correlation between GEBV and TBV; and β_1_: regression coefficient of a regression model of TBV on GEBV.

**Table S4E. SIM5 Accuracy (***r***) and regression coefficient (**$\boldsymbol{\beta}_{\boldsymbol{1}}$**) of genomic estimated breeding value (GEBV) regression model to true breeding value (TBV) for the different genetic methodology schemes tested using the F1-4 validation group.**

| **Rep** | **SC1** | | **SC2_L1** | | **SC2_L2** | | **SC2** | | | **SC3** | | **SC4** | | | **SC5** | |
| --- | --- | --- | --- | --- | --- | --- | --- | --- | --- | --- | --- | --- | --- | --- | --- | --- |
|  | *r* | $\boldsymbol{\beta}_{\boldsymbol{1}}$ | *r* | $\boldsymbol{\beta}_{\boldsymbol{1}}$ | *r* | $\boldsymbol{\beta}_{\boldsymbol{1}}$ | *r* | $\boldsymbol{\beta}_{\boldsymbol{1}}$ | | *r* | $\boldsymbol{\beta}_{\boldsymbol{1}}$ | *r* | $\boldsymbol{\beta}_{\boldsymbol{1}}$ | | *r* | $\boldsymbol{\beta}_{\boldsymbol{1}}$ |
| 1 | 0.46 | 1.02 | 0.43 | 0.97 | 0.46 | 1.22 | 0.46 | | 1.32 | 0.45 | 0.88 | 0.39 | | 1.09 | 0.41 | 0.86 |
| 2 | 0.45 | 1.06 | 0.43 | 1.00 | 0.45 | 1.30 | 0.45 | | 1.40 | 0.44 | 0.91 | 0.43 | | 1.26 | 0.43 | 0.93 |
| 3 | 0.44 | 0.90 | 0.44 | 0.93 | 0.43 | 1.03 | 0.43 | | 1.13 | 0.44 | 0.80 | 0.40 | | 1.03 | 0.39 | 0.78 |
| 4 | 0.48 | 1.15 | 0.46 | 1.12 | 0.48 | 1.36 | 0.48 | | 1.48 | 0.49 | 1.04 | 0.43 | | 1.24 | 0.43 | 0.97 |
| 5 | 0.39 | 0.82 | 0.38 | 0.82 | 0.38 | 0.95 | 0.38 | | 1.03 | 0.39 | 0.74 | 0.37 | | 0.99 | 0.37 | 0.75 |
| 6 | 0.50 | 1.02 | 0.48 | 1.03 | 0.50 | 1.19 | 0.50 | | 1.29 | 0.50 | 0.92 | 0.47 | | 1.11 | 0.48 | 0.93 |
| 7 | 0.46 | 0.96 | 0.43 | 0.96 | 0.46 | 1.13 | 0.46 | | 1.23 | 0.47 | 0.88 | 0.42 | | 1.07 | 0.44 | 0.88 |
| 8 | 0.42 | 0.86 | 0.41 | 0.86 | 0.42 | 1.01 | 0.42 | | 1.10 | 0.41 | 0.83 | 0.37 | | 0.92 | 0.38 | 0.72 |
| 9 | 0.48 | 0.97 | 0.44 | 0.95 | 0.47 | 1.12 | 0.47 | | 1.22 | 0.47 | 0.95 | 0.44 | | 1.12 | 0.43 | 0.84 |
| 10 | 0.47 | 1.12 | 0.45 | 1.09 | 0.48 | 1.36 | 0.48 | | 1.47 | 0.46 | 1.07 | 0.45 | | 1.42 | 0.44 | 1.00 |

SIM5: simulated dataset with heritability explained by the quantitative trait loci (h²_QTL_) = 0.33 and 4,500 QTLs; Rep: simulation replicate ; SC1: single-trait ssGBLUP with a training population represented by purebred and crossbred animals; SC2-L1: multi-trait ssGBLUP with a training population represented by purebred and crossbred animals, and estimation of F1s performance based on results from Line1; SC2-L2: multi-trait ssGBLUP with a training population represented by purebred and crossbred animals, and estimation of F1s performance based on results from Line2; SC3: single-trait WssGBLUP with a training population represented by purebred and crossbred animals; SC4: single-trait WssGBLUP with a training population represented by purebreds and weights estimated from information of crossbred animals; SC5: single-trait WssGBLUP with a training population represented by purebreds; *r*: accuracy represented by Pearson’s correlation between GEBV and TBV; and β_1_: regression coefficient of a regression model of TBV on GEBV.
