# Supplementary material for: Comparing Alternative Single-Step GBLUP Approaches and Training Population Designs for Genomic Evaluation of Crossbred Animals
Source: Front Genet. 2020 Apr 9;11:263. doi: 10.3389/fgene.2020.00263 (PMC7162606; doi:10.3389/fgene.2020.00263)
Supplement: Supplementary file 5 [file Table_5.docx]

**Table S5A. SIM1- Allele frequency correlation across Line1, Line2, and F1 populations.**

| Population pairs | Simulation repetition | | | | | | | | | |
| --- | --- | --- | --- | --- | --- | --- | --- | --- | --- | --- |
|  | 1 | 2 | 3 | 4 | 5 | 6 | 7 | 8 | 9 | 10 |
| Line1 vs. Line2 | 0.45 | 0.45 | 0.45 | 0.45 | 0.47 | 0.43 | 0.47 | 0.47 | 0.48 | 0.46 |
| Line1 vs. F1 (F1-1, F1-2, F1-3, and F1-4) | 0.78 | 0.78 | 0.78 | 0.78 | 0.79 | 0.77 | 0.79 | 0.79 | 0.80 | 0.78 |
| Line2 vs. F1 (F1-1, F1-2, F1-3, and F1-4) | 0.84 | 0.85 | 0.85 | 0.84 | 0.85 | 0.84 | 0.85 | 0.85 | 0.85 | 0.85 |

SIM1: simulated dataset with heritability explained by the quantitative trait loci (h²_QTL_) = 0.

**Table S5B. SIM2- Allele frequency correlation across Line1, Line2, and F1 populations.**

| Population pairs | Simulation repetition | | | | | | | | | |
| --- | --- | --- | --- | --- | --- | --- | --- | --- | --- | --- |
|  | 1 | 2 | 3 | 4 | 5 | 6 | 7 | 8 | 9 | 10 |
| Line1 vs. Line2 | 0.38 | 0.44 | 0.40 | 0.32 | 0.43 | 0.37 | 0.41 | 0.47 | 0.44 | 0.36 |
| Line1 vs. F1 (F1-1, F1-2, F1-3, and F1-4) | 0.74 | 0.78 | 0.75 | 0.71 | 0.77 | 0.74 | 0.75 | 0.78 | 0.77 | 0.73 |
| Line2 vs. F1 (F1-1, F1-2, F1-3, and F1-4) | 0.83 | 0.85 | 0.84 | 0.80 | 0.84 | 0.82 | 0.84 | 0.85 | 0.84 | 0.81 |

SIM2: simulated dataset with heritability explained by the quantitative trait loci (h²_QTL_) = 0.11 and 198 QTLs.

**Table S5C. SIM3- Allele frequency correlation across Line1, Line2, and F1 populations.**

| Population pairs | Simulation repetition | | | | | | | | | |
| --- | --- | --- | --- | --- | --- | --- | --- | --- | --- | --- |
|  | 1 | 2 | 3 | 4 | 5 | 6 | 7 | 8 | 9 | 10 |
| Line1 vs. Line2 | 0.44 | 0.44 | 0.41 | 0.40 | 0.43 | 0.29 | 0.44 | 0.44 | 0.35 | 0.44 |
| Line1 vs. F1 (F1-1, F1-2, F1-3, and F1-4) | 0.76 | 0.77 | 0.76 | 0.75 | 0.76 | 0.68 | 0.77 | 0.78 | 0.72 | 0.78 |
| Line2 vs. F1 (F1-1, F1-2, F1-3, and F1-4) | 0.85 | 0.84 | 0.83 | 0.83 | 0.84 | 0.80 | 0.85 | 0.84 | 0.82 | 0.84 |

SIM3: simulated dataset with heritability explained by the quantitative trait loci (h²_QTL_) = 0.11 and 4,500 QTLs.

**Table S5D. SIM4- Allele frequency correlation across Line1, Line2, and F1 populations.**

| Population pairs | Simulation repetition | | | | | | | | | |
| --- | --- | --- | --- | --- | --- | --- | --- | --- | --- | --- |
|  | 1 | 2 | 3 | 4 | 5 | 6 | 7 | 8 | 9 | 10 |
| Line1 vs. Line2 | 0.23 | 0.34 | 0.24 | 0.35 | 0.31 | 0.33 | 0.32 | 0.30 | 0.30 | 0.28 |
| Line1 vs. F1 (F1-1, F1-2, F1-3, and F1-4) | 0.61 | 0.70 | 0.64 | 0.72 | 0.68 | 0.71 | 0.71 | 0.69 | 0.68 | 0.68 |
| Line2 vs. F1 (F1-1, F1-2, F1-3, and F1-4) | 0.77 | 0.82 | 0.78 | 0.82 | 0.81 | 0.81 | 0.80 | 0.80 | 0.80 | 0.79 |

SIM4: simulated dataset with heritability explained by the quantitative trait loci (h²_QTL_) = 0.33 and 198 QTLs.

**Table S5E. SIM5- Allele frequency correlation across Line1, Line2, and F1 populations.**

| Population pairs | Simulation repetition | | | | | | | | | |
| --- | --- | --- | --- | --- | --- | --- | --- | --- | --- | --- |
|  | 1 | 2 | 3 | 4 | 5 | 6 | 7 | 8 | 9 | 10 |
| Line1 vs. Line2 | 0.25 | 0.31 | 0.25 | 0.38 | 0.39 | 0.33 | 0.31 | 0.35 | 0.28 | 0.31 |
| Line1 vs. F1 (F1-1, F1-2, F1-3, and F1-4) | 0.65 | 0.68 | 0.65 | 0.73 | 0.74 | 0.70 | 0.67 | 0.71 | 0.67 | 0.69 |
| Line2 vs. F1 (F1-1, F1-2, F1-3, and F1-4) | 0.79 | 0.81 | 0.78 | 0.83 | 0.83 | 0.82 | 0.81 | 0.81 | 0.80 | 0.81 |

SIM5: simulated dataset with heritability explained by the quantitative trait loci (h²_QTL_) = 0.33 and 4,500 QTLs.
