# Supplementary material for: Comparing Alternative Single-Step GBLUP Approaches and Training Population Designs for Genomic Evaluation of Crossbred Animals
Source: Front Genet. 2020 Apr 9;11:263. doi: 10.3389/fgene.2020.00263 (PMC7162606; doi:10.3389/fgene.2020.00263)
Supplement: Supplementary file 6 [file Table_6.docx]

**Table S6A. SIM1 Accuracy (***r***) and regression coefficient (**$\boldsymbol{\beta}_{\boldsymbol{1}}$**) of true breeding value (TBV) on genomic estimated breeding value (GEBV) for the single-trait ssGBLUP with a balanced training population represented by purebred and crossbred animals (total 10,000 individuals in training population- SC1), and the single-trait WssGBLUP with a training population represented by purebred and crossbred animals (total 10,000 individuals- SC3) using the F1-3 validation group.**

| **Rep** | **SC1** | | **SC3** | |
| --- | --- | --- | --- | --- |
|  | *r* | $\boldsymbol{\beta}_{\boldsymbol{1}}$ | **r²** | $\boldsymbol{\beta}_{\boldsymbol{1}}$ |
| 1 | 0.15 | 0.40 | 0.15 | 0.34 |
| 2 | 0.10 | 0.31 | 0.11 | 0.28 |
| 3 | 0.10 | 0.26 | 0.09 | 0.20 |
| 4 | 0.18 | 0.50 | 0.16 | 0.38 |
| 5 | 0.07 | 0.23 | 0.07 | 0.20 |
| 6 | 0.07 | 0.20 | 0.05 | 0.14 |
| 7 | 0.10 | 0.28 | 0.09 | 0.22 |
| 8 | 0.17 | 0.52 | 0.16 | 0.44 |
| 9 | 0.21 | 0.64 | 0.20 | 0.54 |
| 10 | 0.14 | 0.36 | 0.11 | 0.24 |

SIM1: simulated dataset with heritability explained by the quantitative trait loci (h²_QTL_) = 0; Rep: simulation replicate.

**Table S6B. SIM2 Accuracy (***r***) and regression coefficient (**$\boldsymbol{\beta}_{\boldsymbol{1}}$**) of true breeding value (TBV) on genomic estimated breeding value (GEBV) for the single-trait ssGBLUP with a balanced training population represented by purebred and crossbred animals (total 10,000 individuals in training population- SC1), and the single-trait WssGBLUP with a training population represented by purebred and crossbred animals (total 10,000 individuals- SC3) using the F1-3 validation group.**

| **Rep** | **SC1** | | **SC3** | |
| --- | --- | --- | --- | --- |
|  | *r* | $\boldsymbol{\beta}_{\boldsymbol{1}}$ | *r* | $\boldsymbol{\beta}_{\boldsymbol{1}}$ |
| 1 | 0.12 | 0.33 | 0.12 | 0.29 |
| 2 | 0.26 | 0.70 | 0.25 | 0.58 |
| 3 | 0.25 | 0.63 | 0.26 | 0.55 |
| 4 | 0.14 | 0.35 | 0.14 | 0.30 |
| 5 | 0.27 | 0.90 | 0.26 | 0.76 |
| 6 | 0.14 | 0.40 | 0.14 | 0.34 |
| 7 | 0.22 | 0.58 | 0.23 | 0.51 |
| 8 | 0.26 | 0.70 | 0.24 | 0.56 |
| 9 | 0.27 | 0.74 | 0.26 | 0.61 |
| 10 | 0.30 | 0.77 | 0.29 | 0.64 |

SIM2: simulated dataset with heritability explained by the quantitative trait loci (h²_QTL_) = 0.11 and 198 QTLs; Rep: simulation replicate.

**Table S6C. SIM3 Accuracy (***r***) and regression coefficient (**$\boldsymbol{\beta}_{\boldsymbol{1}}$**) of true breeding value (TBV) on genomic estimated breeding value (GEBV) for the single-trait ssGBLUP with a balanced training population represented by purebred and crossbred animals (total 10,000 individuals in training population- SC1), and the single-trait WssGBLUP with a training population represented by purebred and crossbred animals (total 10,000 individuals- SC3) using the F1-3 validation group.**

| **Rep** | **SC1** | | **SC3** | |
| --- | --- | --- | --- | --- |
|  | *r* | $\boldsymbol{\beta}_{\boldsymbol{1}}$ | *r* | $\boldsymbol{\beta}_{\boldsymbol{1}}$ |
| 1 | 0.27 | 0.75 | 0.25 | 0.60 |
| 2 | 0.28 | 0.74 | 0.26 | 0.60 |
| 3 | 0.30 | 0.76 | 0.28 | 0.61 |
| 4 | 0.26 | 0.67 | 0.23 | 0.53 |
| 5 | 0.25 | 0.68 | 0.23 | 0.55 |
| 6 | 0.23 | 0.56 | 0.20 | 0.42 |
| 7 | 0.14 | 0.36 | 0.13 | 0.28 |
| 8 | 0.21 | 0.57 | 0.18 | 0.44 |
| 9 | 0.31 | 0.77 | 0.29 | 0.64 |
| 10 | 0.29 | 0.78 | 0.29 | 0.67 |

SIM3: simulated dataset with heritability explained by the quantitative trait loci (h²_QTL_) = 0.11 and 4,500 QTLs; Rep: simulation replicate.

**Table S6D. SIM4 Accuracy (***r***) and regression coefficient (**$\boldsymbol{\beta}_{\boldsymbol{1}}$**) of true breeding value (TBV) on genomic estimated breeding value (GEBV) for the single-trait ssGBLUP with a balanced training population represented by purebred and crossbred animals (total 10,000 individuals in training population- SC1), and the single-trait WssGBLUP with a training population represented by purebred and crossbred animals (total 10,000 individuals- SC3) using the F1-3 validation group.**

| **Rep** | **SC1** | | **SC3** | |
| --- | --- | --- | --- | --- |
|  | *r* | $\boldsymbol{\beta}_{\boldsymbol{1}}$ | *r* | $\boldsymbol{\beta}_{\boldsymbol{1}}$ |
| 1 | 0.29 | 0.58 | 0.31 | 0.53 |
| 2 | 0.40 | 0.86 | 0.43 | 0.78 |
| 3 | 0.38 | 0.75 | 0.40 | 0.69 |
| 4 | 0.33 | 0.70 | 0.36 | 0.65 |
| 5 | 0.34 | 0.66 | 0.34 | 0.55 |
| 6 | 0.31 | 0.63 | 0.34 | 0.59 |
| 7 | 0.36 | 0.71 | 0.38 | 0.62 |
| 8 | 0.27 | 0.55 | 0.30 | 0.50 |
| 9 | 0.25 | 0.55 | 0.27 | 0.50 |
| 10 | 0.27 | 0.58 | 0.29 | 0.52 |

SIM4: simulated dataset with heritability explained by the quantitative trait loci (h²_QTL_) = 0.33 and 198 QTLs; Rep: simulation replicate.

**Table S6E. SIM5 Accuracy (***r***) and regression coefficient (**$\boldsymbol{\beta}_{\boldsymbol{1}}$**) of true breeding value (TBV) on genomic estimated breeding value (GEBV) for the single-trait ssGBLUP with a balanced training population represented by purebred and crossbred animals (total 10,000 individuals in training population- SC1), and the single-trait WssGBLUP with a training population represented by purebred and crossbred animals (total 10,000 individuals- SC3) using the F1-3 validation group.**

| **Rep** | **SC1** | | **SC3** | |
| --- | --- | --- | --- | --- |
|  | *r* | $\boldsymbol{\beta}_{\boldsymbol{1}}$ | *r* | $\boldsymbol{\beta}_{\boldsymbol{1}}$ |
| 1 | 0.47 | 1.12 | 0.46 | 0.97 |
| 2 | 0.36 | 0.93 | 0.36 | 0.79 |
| 3 | 0.45 | 1.06 | 0.44 | 0.89 |
| 4 | 0.47 | 1.02 | 0.46 | 0.88 |
| 5 | 0.46 | 1.10 | 0.45 | 0.96 |
| 6 | 0.47 | 1.04 | 0.48 | 0.92 |
| 7 | 0.44 | 0.95 | 0.43 | 0.82 |
| 8 | 0.39 | 0.84 | 0.37 | 0.72 |
| 9 | 0.43 | 1.01 | 0.42 | 0.86 |
| 10 | 0.37 | 0.83 | 0.37 | 0.72 |

SIM5: simulated dataset with heritability explained by the quantitative trait loci (h²_QTL_) = 0.33 and 4,500 QTLs; Rep: simulation replicate.
